# Growth Factor PDGF-BB Stimulates Cultured Cardiomyocytes to Synthesize the Extracellular Matrix Component Hyaluronan

**DOI:** 10.1371/journal.pone.0014393

**Published:** 2010-12-21

**Authors:** Urban Hellman, Linus Malm, Li-Ping Ma, Göran Larsson, Stellan Mörner, Michael Fu, Anna Engström-Laurent, Anders Waldenström

**Affiliations:** 1 Department of Public Health and Clinical Medicine/Medicine, Umeå University, Umeå, Sweden; 2 Department of Medical Biosciences/Medical and Clinical Genetics, Heart Center, Umeå University Hospital, Umeå University, Umeå, Sweden; 3 Department of Medical Biochemistry and Biophysics, Umeå University, Umeå, Sweden; 4 Department of Cardiology, Changhai Hospital, The Second Military Medical University, Shanghai, China; 5 Wallenberg Laboratory, Department of Molecular and Clinical Medicine, Institute of Medicine, Sahlgrenska Academy, Gothenburg University, Gothenburg, Sweden; Victor Chang Cardiac Research Institute, Australia

## Abstract

**Background:**

Hyaluronan (HA) is a glycosaminoglycan located in the interstitial space which is essential for both structural and cell regulatory functions in connective tissue. We have previously shown that HA synthesis is up-regulated in a rat model of experimental cardiac hypertrophy and that cardiac tissue utilizes two different HA synthases in the hypertrophic process. Cardiomyocytes and fibroblasts are two major cell types in heart tissue. The fibroblasts are known to produce HA, but it has been unclear if cardiomyocytes share the same feature, and whether or not the different HA synthases are activated in the different cell types.

**Methodology/Principal Findings:**

This study shows, for the first time that cardiomyocytes can produce HA. Cardiomyocytes (HL-1) and fibroblasts (NIH 3T3) were cultivated in absence or presence of the growth factors FGF2, PDGF-BB and TGFB2. HA concentration was quantified by ELISA, and the size of HA was estimated using dynamic light scattering. Cardiomyocytes synthesized HA but only when stimulated by PDGF-BB, whereas fibroblasts synthesized HA without addition of growth factors as well as when stimulated by any of the three growth factors. When fibroblasts were stimulated by the growth factors, reverse dose dependence was observed, where the highest dose induced the least amount of HA. With the exception of TGFB2, a trend of reverse dose dependence of HA size was also observed.

**Conclusions/Significance:**

Co-cultivation of cardiomyocytes and fibroblasts (80%/20%) increased HA concentration far more that can be explained by HA synthesis by the two cell types separately, revealing a crosstalk between cardiomyocytes and fibroblasts that induces HA synthesis. We conclude that dynamic changes of the myocardium, such as in cardiac hypertrophy, do not depend on the cardiomyocyte alone, but are achieved when both cardiomyocytes and fibroblasts are present.

## Introduction

Hyaluronan (HA) is a prominent structural component in the extracellular matrix (ECM), and is distributed ubiquitously in adult vertebrate tissues. HA is a nonsulphated, linear, high molecular weight glycosaminoglycan (GAG) composed of repeating disaccharides, D-glucuronic acid and *N*-acetyl-D-glucosamine [1]. HA has unique hydrodynamic properties, functioning as a biological lubricant in joints during movement and providing resilience in static conditions [2]. HA is synthesized by membrane-bound synthases (HAS) at the inner surface of the plasma membrane. The growing polymer is extruded through the membrane to the outside of the cell as it is being synthesized. Three mammalian HAS's have been identified, HAS1, HAS2 and HAS3, which differ in catalytic activity as well as in product size [3]. Several growth factors regulate HA biosynthesis and the induction of the three different HAS's [4], [5], [6]. Different responses in HA biosynthesis are depending on growth factor and cell type. In its native state, the HA molecule (n-HA) can be composed of 2000–25,000 of repeating disaccharides, with molecular weights ranging from 10^6^ to 10^7^ Da and with an extended length of 2–25 µm [3], [7]. Via receptors on the cell surface HA has also a signaling property. These receptors include, for example, CD44 and RHAMM (receptor for hyaluronan-mediated motility), [8], [9], [10]. HA-receptor interactions mediate at least three physiological processes: signal transduction, HA internalization and assembly of extracellular matrices [11], [12]. The length of the HA molecule affects its signaling properties. For example, n-HA is anti-angiogenic while oligo-HA (o-HA) is pro-angiogenic [13], [14]. Large and small HA molecules interact differently with extracellular and cell-surface HA-binding proteins[15]. Depending on its size, HA has been shown to be involved in many processes including wound healing and scarless fetal healing [16], [17]. High concentrations of HA in many embryonic tissues correlate with cell migration and proliferation. In fact, similarities between developing tissues and tumor tissues are demonstrated by higher concentrations of HA in many malignant tumors than in corresponding benign tumors or normal tissues [18], [19], [20]. The function of HA in the heart has so far not been thoroughly investigated. However, absence of HA causes abnormalities in heart and blood vessel development, resulting in an embryonic lethal phenotype, showing the importance of HA in heart formation [21], [22]. HA synthesis has also been shown to be up-regulated in experimental myocardial infarction and myocarditis [23], as well as in an experimental rat model of cardiac hypertrophy [24] where the increased amount of HA was linked to different HA synthases in the hypertrophic process. Fibroblasts are well known synthesizers of HA, while cardiomyocytes have not been studied in this respect. It is still unknown which cell types in the heart are involved in these processes and if the two HASs in the hypertrophic process are responsible for different sizes of HA, hence synthesized for different purposes. To further investigate the role of HA in the heart, cultured cardiomyocytes and fibroblasts were studied separately. The aims of the study were the following: a) to determine whether cardiomyocytes are capable of synthesizing HA, using three different growth factors, well known to stimulate HA synthesis in fibroblasts, fibroblast growth factor-2 (FGF2), platelet-derived growth factor-BB (PDGF-BB) and transforming growth factor-β2 (TGFB2) [4], [5], b) to find out if there is a cell signaling between fibroblasts and cardiomyocytes leading to HA synthesis and in what manner the signal is transduced, and c) test to determine if the size of the HA produced can be related to biological effects.

## Material and Methods

### Cell culture

HL-1, a cell line derived from adult mouse heart, displaying phenotypic features typical of adult cardiomyocytes [25], was obtained from Dr. W.C. Claycomb (Louisiana State University Medical Center, New Orleans). Cardiomyocytes plated in T-75 flasks coated with fibronectin (Sigma)-gelatin (Fisher Scientific) were maintained in Claycomb Medium (JRH. Biosciences) supplemented with 10% fetal bovine serum (JRH. Biosciences), 0.1 mM norepinephrine [consisting of 10 mM norepinephrine (Sigma-Aldrich) dissolved in 30 mM ascorbic acid (Sigma)], 2 mM L-glutamine (Life Technologies), and 100 U/mL penicillin, 100 µg/mL streptomycin (Life Technologies). During culture, the medium was changed routinely every 24 h. Fibroblasts, NIH 3T3 cells [26] (ATCC CRL-1658, LGC Standards AB) were cultured and passaged following the standard procedure in Dulbecco's modified Eagle's medium (DMEM, Fisher Scientific) containing 10% calf serum (JRH. Biosciences), 2 mM L-glutamine, 100 U/mL penicillin, and 100 µg/mL streptomycin.

HL-1 and NIH3T3 cells were passaged twice per week by trypsinization (Trypsin- EDTA, Life Technologies). All cultures were kept in an atmosphere of 95% air-5% CO_2_, 37°C and at a relative humidity of approximately 95%.

### Pretreatments of cells

NIH 3T3 cells -alone and HL-1cells -alone were plated at a concentration of 0.9×10^6^ cells/well into 6-well plates. In a monolayer co-culture of HL-1 with NIH 3T3, 0.72×10^6^ HL-1 cells/well were mixed with 0.18×10^6^/well of NIH 3T3 cells to a 6-well plate. After 72 h, cells were grown to confluence and all media were replaced with serum-free and antibiotic-free media for 24 h. In the treatment groups, cells were then stimulated with three different growth factors, known to induce HA synthesis in cultured fibroblasts, dissolved in serum-free and antibiotic-free media, FGF2 (5 ng/mL, 10 ng/mL), PDGF-BB (50 ng/mL, 100 ng/mL) and TGFB2 (5 ng/mL, 10 ng/mL) (Biosource, Invitrogen), respectively, six replicates of each. Plates with no growth factor addition were used as controls. After 24 h, the media from each growth condition was collected and the cells were then washed with ice-cold phosphate buffered saline (PBS) (without Ca & Mg, Fisher Scientific), and subsequently harvested by trypsination or scraping and placed in RNAlater (Qiagen).

### Quantitative analysis of hyaluronan

The six replicates of media were diluted 20–100 times in PBS and analyzed for HA content in duplicate by an enzyme-linked binding protein assay (Corgenix) according to the manufacturer's instructions. Absorbance was read at 450 nm with correction at 650 nm on a spectrophotometer (Multiscan Ascent, Thermo Labsystems). Data were expressed as mean±SD.

### RNA preparation

For each cell group, total RNA was isolated from three wells using the RNeasy Fibrous Tissue Kit (Qiagen). The concentration of the RNA was measured in a NanoDrop ND-1000 Spectrophotometer (NanoDrop Technologies Inc.) and the integrity of the RNA was analyzed in a 2100 Bioanalyser (Agilent Technologies Inc.). Omniscript RT Kit (Qiagen) was used to synthesize cDNA.

### Real-time polymerase chain reaction

Relative quantification of gene expression changes was performed using an Applied Biosystems Prism 7900HT Sequence Detection System according to the manufacturer's specifications (Applied Biosystems). Mouse cDNA-specific TaqMan Gene Expression Assays for HAS1, HAS2 and HAS3 from Applied Biosystems were used. The mouse D-glyceraldehyde-3-phosphate dehydrogenase (*Gapdh*) gene was used as an endogenous control (part number 4352338E; Applied Biosystems). All samples were run in triplicate, and amplification was analyzed using Applied Biosystems Prism Sequence Detection Software version 2.3.

### Hyaluronan purification

Hyaluronan samples were purified from cell media of cultured cardiomyocytes and fibroblasts. Four mL of cell media were concentrated to 50 µL using 10 kDa cut-off Ultra Cell filter-unit (Millipore) by 15 min centrifugation at 4,000×g using a centrifuge (Sigma 4K10) equipped with a swing-out rotor. Concentrated samples were diluted with 1 mL of 100 mM CaCl_2_ in 20 mM TRIS-HCl buffer at pH 7.4. A total digestion of proteins, present in the growth medium, was utilized using Pronase (Boehringer Mannheim) at a concentration of 2 mg/mL in each sample. The protein digestion was allowed to continue for 72 h at 40°C and subsequently quenched by increasing the temperature to 100°C for 3 minutes. Digested peptides and amino acids, as well as CaCl_2_, were removed using a 10 kDa cut-off Ultra Cell filter-unit by 3×15 min centrifugation at 4,000×g. After each concentration step the samples were diluted with 1 mL of 20 mM TRIS-HCl adjusted to pH 7.4.

Desalted HA samples were loaded on a DEAE-FF anion-exchange column with a bead volume of 1 mL (GE-Life Science) using a peristaltic pump (Bio-Rad) operating at 1 mL/min. The column was washed with 5 and 8 column volumes of 20 mM TRIS-HCl pH 7.4 containing 0 M or 0.2 M NaCl, respectively. HA was eluted from the column with 8 column volumes of 0.4 M NaCl 20 mM TRIS-HCl pH 7.4.

The eluted fraction was collected and concentrated to approximately 1.5 mL as described above. The volume of the concentrated samples was further reduced to 30 µL using a 0.5 mL 10 kDa cut-off Ultra Cell filter-unit using a Microfuge 18 tabletop centrifuge (Beckman Coulter) at 14,000 rpm. The buffer was changed by 3 steps of 15 min centrifugation followed by dilution with 0.5 mL of 100 mM NaCl in D_2_O using the same concentration device. In all concentration steps the filters were washed with 0.1 M NaOH followed by careful rinsing with the appropriate buffer to avoid contamination of glycerol in the samples.

The purity of the samples was controlled by SDS-page, for detection of residual proteins after Pronase digestion, and by NMR to detect any other impurities, such as lipids and DNA.

### Dynamic light scattering size analysis of hyaluronan

The size of HA synthesized by fibroblasts, cardiomyocytes or a co-culture of the two cells types were estimated by DLS. The Z-average value, which is the mean hydrodynamic diameter weighted against the intensity of the DLS signal, was used to estimate the mean hydrodynamic size of the different HA molecules. Due to the hygroscopic properties of HA, the hydrodynamic diameter of HA is highly dependent on the buffer and salt conditions used in the DLS measurements. Therefore, the hydrodynamic diameters of HA purified from cell media were compared with those of HA with known molecular weights of 70 kDa, 450 kDa and 2420 kDa (Hyalose, L.L.C.) recorded at identical conditions.

Each HA sample was intensively shaken at 4°C for 20 minutes, to dissolve possible entanglements. To sediment remaining entanglements, the samples were centrifuged for 20 minutes by a Microfuge 18 tabletop centrifuge (Beckman Coulter) at 14,000 rpm, 50 µL of sample was added into a disposable low volume cuvette with a 10 mm path length. DLS measurements were conducted at 20°C using a Nano Zetasizer (Malvern Instruments) equipped with a HeNe-laser with a wave length of 633 nm. Before each measurement the temperature was allowed to equilibrate for 10 minutes. The back scattered light was detected at an angle of 173 degrees. For each cell condition the light scattering was measured for 200 seconds with 10 replicate measurements. The DLS data was analyzed using the Dispersion Technology Software v.5.10 (Malvern). Z-average, the mean intensity weighted diameter, was collected for each HA sample. Mean values and standard deviations from each measurement were calculated and compared using GrapPad Prism v.5. Outliers in Z-average were detected and rejected by calculation of the Dixon's *Q* ratio, using *P* = 0.05 [27].

### Crosstalk between cardiomyocytes and fibroblasts

After 24 h incubation, media from cardiomyocytes, fibroblasts and co-cultured cardiomyocytes and fibroblasts were transferred to both cardiomyocyte and fibroblast cultures. After additional 24 h incubation, media were collected and the cells were harvested by scraping and placed in RNAlater. Concentration of HA in media from cardiomyocytes and fibroblasts were measured as described above. To distinguish between extracellular signaling through microvesicles and exocytosis of soluble molecules, media that induced increased HA synthesis in cells was centrifuged to remove cell debris, 3,000×g for 20 minutes at 4°C, repeated three times, followed by 10,000×g for 20 minutes at 4°C, repeated three times. The acquired supernatant was ultracentrifuged at 130,000×g (49,000 rpm) for 2 h at 4°C in a MLS-50 rotor and a Beckman Optima™ MAX-E Ultracentrifuge (Beckman Coulter) to separate pellet and media supernatant containing soluble molecules. Cells were then incubated with pellet dissolved in new media or with media supernatant. The concentration of HA in media was measured and compared to controls incubated with unused media. Gene expression in cells (n = 3, except fibroblasts incubated with media supernatant, where n = 2) was analyzed with Illumina Beadstation (Illumina, San Diego).

To investigate the presence of HA interaction with cardiomyocytes, n-HA, 10 µg/mL, 800–1200 kDa (Select-HA™ 1000) or o-HA, 50 µg/mL, 6-mer (HYA-OLIGO6EF-1) (Hyalose, L.L.C.) was added to cardiomyocytes in Claycomb media, followed by 24 h incubation. The cells were harvested by scraping and placed in RNAlater.

Differential gene expression, analyzed with Illumina Beadstation, was used to detect intracellular signaling resulting in changed transcription, compared to control cells (n = 2 in each group).

### Microarray gene expression

Aliquots of total RNA were converted to biotinylated double-stranded cRNA according to the specifications of the Illumina Totalprep RNA Amplification Kit (Ambion). The labelled cRNA samples were hybridized to MouseRef-8 Expression Beadchip (Illumina), incubated with streptavidin-Cy3 and scanned on the Illumina Beadstation GX.

### Data analysis of gene expression microarrays

To determine differentially expressed genes microarray data were analyzed using gene expression module in Illumina Beadstudio software, version 3.3.7. Intensity data was normalized using the Beadstudio cubic spline algorithm. Significant differential expression was calculated using the Beadstudio software by applying multiple testing corrections using Benjamini and Hochberg False Discovery Rate (FDR) [28], [29]. All data is MIAME compliant and is available through NCBIs Gene Expression Omnibus (GEO) (GEO Series accession number GSE21675 and GSE21676).

The gene expression fold change of the stimulated cells was calculated as the average signal value relative to the average signal value for the control cells. A significant up-regulation was defined as a foldchange ≥1.5 and a significant down-regulation was defined as foldchange ≤0.67. Statistical significance was set at P<0.05. To avoid selecting genes with high foldchange due to low signal intensity a minimum signal intensity value was utilized. For up-regulated genes the signal intensity was set at >50 in the stimulated cell group. For down-regulated genes the signal intensity was set at >50 in the control cell group.

## Results

### Quantitative analysis of hyaluronan in cell media

**Figure 1:**
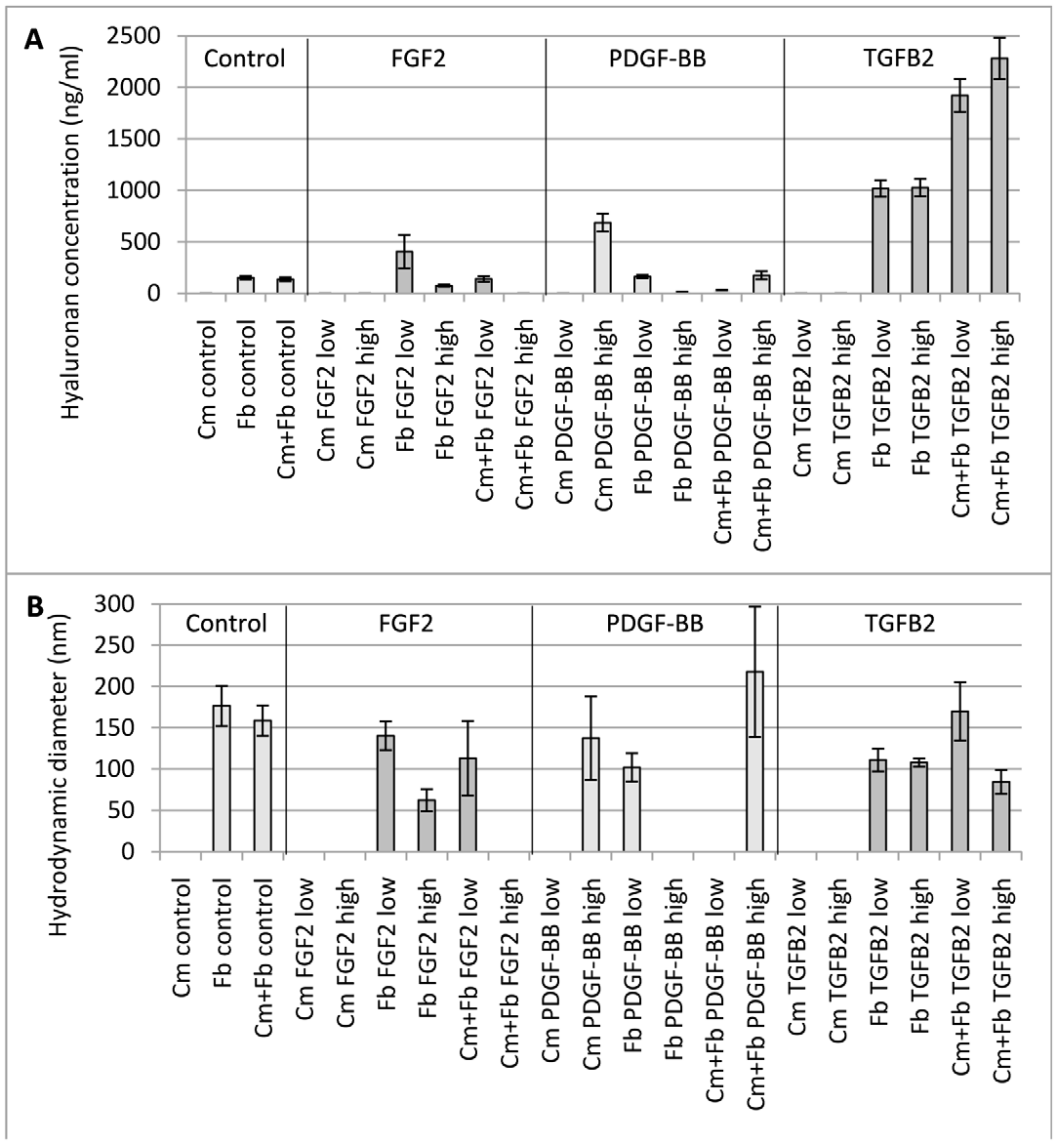
HA concentration and size in cell media. Cultured cardiomyocytes, fibroblasts and co-cultured cells (80%/20%) was stimulated with growth factors (FGF2, fibroblast growth factor-2, PDGF-BB, platelet-derived growth factor-BB and TGFB2, transforming growth factor-β2). “Low” indicates a growth factor concentration in the media of 50 ng/mL for PDGF-BB and 5 ng/mL for FGF2 and TGFB2. “High” indicates a growth factor concentration in the media of 100 ng/mL for PDGF-BB and 10 ng/mL for FGF2 and TGFB2. HA concentration was measured with an enzyme-linked binding protein assay. HA size was estimated with DLS.

HA concentration in cultured cardiomyocytes and fibroblasts was quantified to determine HA synthesis in response to stimulation by the different growth factors FGF2, PDGF-BB and TGFB2. The only growth factor that caused cardiomyocytes to synthesize HA was PDGF-BB at 100 ng/mL in the media. Fibroblasts on the other hand synthesize HA with or without any addition of growth factors. The addition of growth factors showed that FGF2 and PDGF-BB at higher concentrations (10 and 100 ng/mL) induced a lower concentration of HA than at lower concentration (5 and 50 ng/mL). TGFB2 induced the highest concentration of HA but no difference was seen in relation to its concentration (Figure 1A).

Co-culturing cardiomyocytes and fibroblasts resulted in increased HA concentration compared to cells cultured separately, especially when taking into account the lower amount of fibroblasts in the co-culture.

In contrast, FGF2 and PDGF-BB stimulated co-cultured cells resulted in lower HA concentration in the media compared to cells cultured alone. Media from co-cultured cells stimulated with TGFB2 contained twice as much HA as in TGFB2 stimulated fibroblasts (Figure 1A).

### Real-time polymerase chain reaction analysis of hyaluronan synthases

Concerning assessment of expression of *Has* genes, the endogenous control *Gapdh*, used in real-time PCR analysis to normalize for differences in the amount of total RNA added, were in some cell/growth factor combinations not at a consistent level between samples and controls. Therefore, the *β-actin* and *36B4* genes were also tested as endogenous controls but with the same result. For this reason the real-time PCR results have only been used as a detection of expression of the different *Has's* without the possibility to calculate foldchange and significance of changes in expression (Table 1).

**Table 1:** HA synthases detected with real-time PCR.

Cell type/growth factor	*Has1*	*Has2*	*Has3*
Cardiomyocytes/PDGF-BB	n.d.	d	d
Fibroblasts/PDGF-BB	n.d.	d	n.d.
Fibroblasts/FGF2	n.d.	d	n.d.
Fibroblasts/TGFB2	n.d.	d	d
Co-cultured cells/PDGF-BB	n.d.	d	n.d.
Co-cultured cells/FGF2	n.d.	d	n.d.
Co-cultured cells/TGFB2	n.d.	d	n.d.

d., detected. n.d.; not detected.

### Dynamic light scattering *size analysis of hyaluronan*

**Figure 2:**
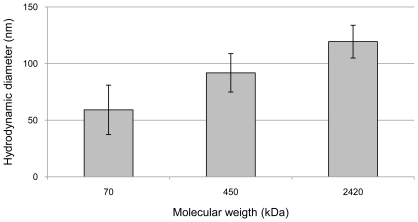
Size measurement of HA with known molecular weigths. The hydrodynamic diameters of HA with known molecular weights of 70 kDa, 450 kDa and 2420 kDa.

The size of HA synthesized by fibroblasts, cardiomyocytes or co-culture of the two cell types was estimated by dynamic light scattering (DLS). The Z-average value of HA with known molecular weights of 70 kDa, 450 kDa and 2420 kDa (Hyalose, L.L.C.) gave Z-average values of 60±20 nm, 90±15 nm and 120±15 nm, respectively (Figure 2).

Cardiomyocytes produced HA with a diameter of 135±50 nm when stimulated with a high concentration of PDGF-BB. Under other conditions cardiomyocytes did not produce any detectable levels of HA (Figure 1B).

Non-stimulated fibroblasts produced HA with a hydrodynamic diameter of 175±25 nm. Upon stimulation by low levels of FGF2, fibroblasts released HA with a diameter of 140±20 nm, whereas fibroblasts cultured with high levels of FGF2 produced HA with a diameter of 60±15 nm. Fibroblasts stimulated by the low dose of PDGF-BB produce HA with a diameter of 100±15 nm. When the fibroblasts were stimulated by a high dose of PDGF-BB the concentration of HA became too low to ensure reliable DLS measurements. The different concentrations of TGFB2 showed little effect on the size of produced HA, with a hydrodynamic diameter of 110±15 nm and 110±5 nm for the low and high concentration of this growth factor, respectively (Figure 1B).

When cardiomyocytes and fibroblasts were co-cultured without any growth factors they secreted HA with a diameter of 160±20 nm. The addition of low levels of FGF2 induced HA of 115±45 nm, whereas the high level of FGF2 yielded no detectable HA. Low levels of PDGF-BB did not induce a HA concentration high enough for reliable DLS measurements, whereas a high dose of PDGF-BB yielded HA with a size of 220±80 nm. When a low dose of TGFB2 was added to co-cultured cells, HA with a size of 170±35 nm was produced, whereas the high concentration of TGFB2 resulted in HA with a diameter of 85±15 nm (Figure 1B).

In four of the HA size measurements a larger standard deviation of HA were observed. This was caused by a larger range of sizes of the HA molecules produced under these specific conditions, and not caused by impurities or HA aggregates.

In one of the fibroblast control samples one light scattering measurements was identified as an outlier by Dixons *Q* test (*P*- 0.05) and excluded from the calculated mean value. The identified outlier was most likely caused small amounts of HA aggregates.

### Crosstalk between cardiomyocytes and fibroblasts

**Figure 3:**
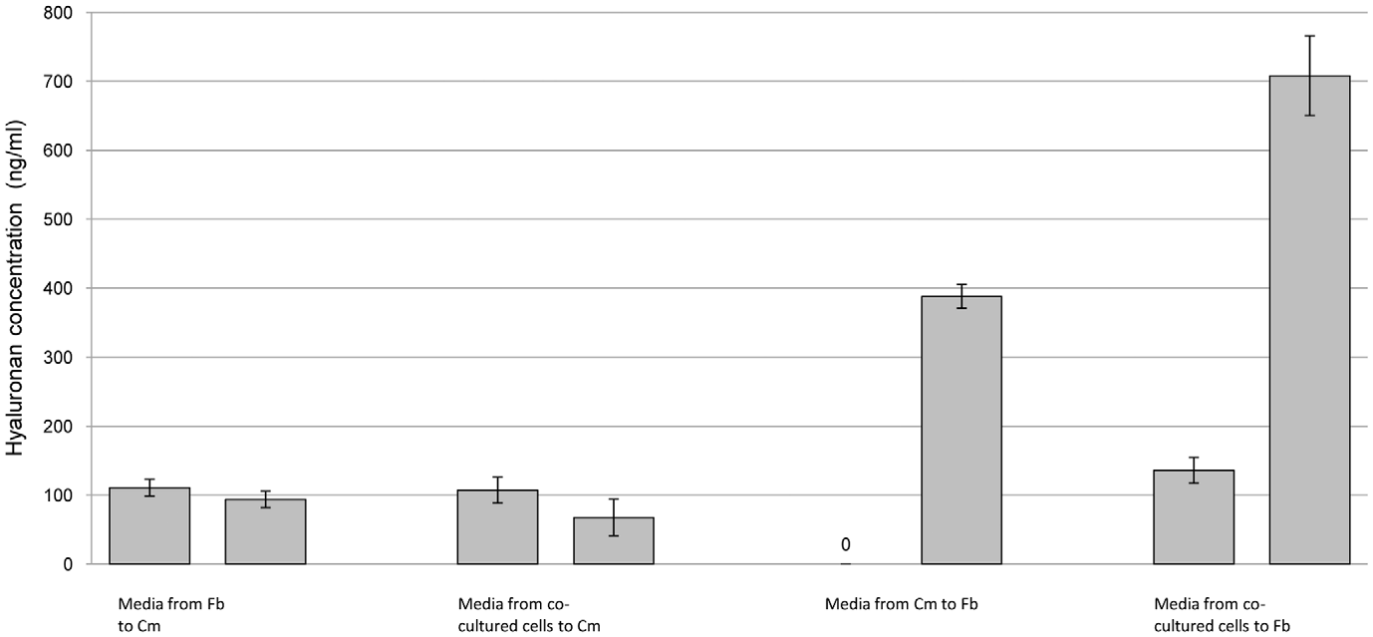
HA concentration in media transferred between different cell types. HA concentration in media was measured before and after transfer between different cell types. After 24 h Claycomb media was transferred from Cm and co-cultured cells to Fb and DMEM media was transferred from Fb to Cm and co-cultured cells. Cm, cardiomyocytes; Fb, fibroblasts.

**Figure 4:**
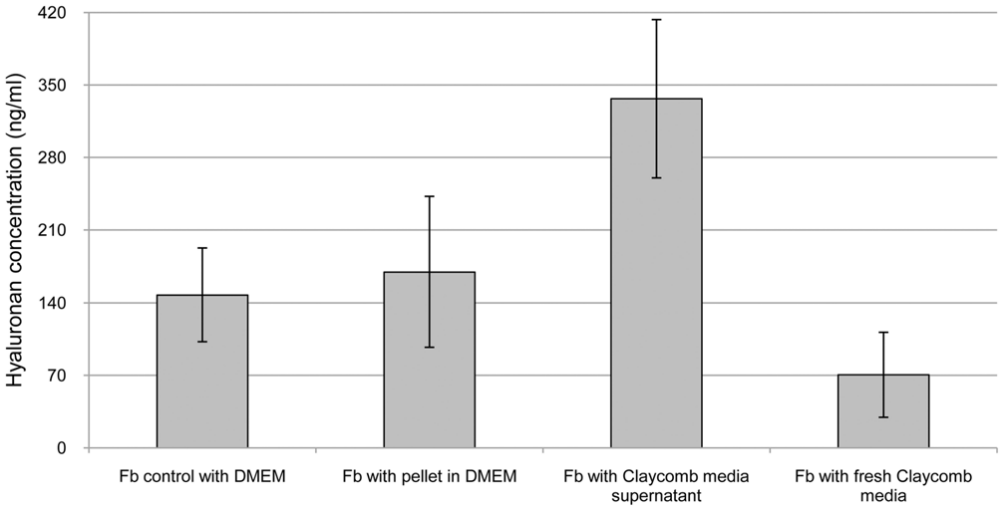
HA concentration in ultracentrifuged media. Claycomb media that induced increased HA synthesis in Fb was ultracentrifuged. Pellet after ultracentrifugation was dissolved in DMEM. Fb was incubated for 24 h with pellet in DMEM and Claycomb media supernatant. Fb was also incubated with fresh, unused Claycomb media. Fb, fibroblasts.

The presence of signaling crosstalk between cultured cardiomyocytes and fibroblasts that induces HA synthesis was elucidated by quantification of HA and gene expression analysis of both cell types. Measurements of HA concentration in cell media and gene expression analysis were used to elucidate the existence of signaling crosstalk between cardiomyocytes and fibroblasts. Three general ways of mediating signaling crosstalk between cells were considered. Direct cell contact, extracellular signaling transferred by microvesicles released into media [30] or extracellular signaling transferred through exocytosis of soluble molecules into media. Synthesis of HA by fibroblasts increased after incubation with Claycomb media from either co-cultured cells or cardiomyocytes alone, compared to both fibroblasts incubated in DMEM and fibroblasts incubated in fresh, unused Claycomb media (Figure 3,4). A lower HA concentration was obtained after incubation of fibroblasts with fresh, unused Claycomb media. Fibroblasts which were incubated with pellet from ultracentrifugation of Claycomb media from cardiomyocytes, dissolved in DMEM showed similar HA concentration to fibroblast controls while fibroblasts incubated in the supernatant of ultracentrifuged Claycomb media from cardiomyocytes demonstrated a two-fold increase in HA concentration (Figure 4). Cardiomyocytes showed no synthesis of HA irrespective of media added (Figure 3).

Gene expression analysis of fibroblasts incubated for 24 h with Claycomb media transferred from cardiomyocytes, resulted in 333 differentially expressed genes compared to fibroblasts incubated with unused Claycomb media (Table 2) (Table S1). The same analysis of fibroblasts incubated for 24 h with ultracentrifuged Claycomb media supernatant transferred from cardiomyocytes, resulted in 96 differentially expressed genes (Table 2) (Table S2). In both cases the *Tgfb2* gene was up-regulated 3-fold. Data and information is available through NCBI's GEO (accession number GSE21675).

**Table 2:** Number of differentially expressed genes after each filtering step.

Filtering step	Cm with oHA		Cm with nHA		Fb with Cm media		Fb with Cm media sup.	
Differential	554		493		1384		501	
*P*-value<0.05	288↑	266↓	69↑	424↓	695↑	689↓	201↑	300↓
	↓		↓		↓		↓	
FDR	130		105		400		102	
	75↑	55↓	4↑	101↓	213↑	187↓	21↑	81↓
	↓		↓		↓		↓	
Detection	111		45		335		96	
*P*-value<0.05	68↑	43↓	3↑	42↓	177↑	158↓	21↑	75↓
	↓		↓		↓		↓	
Foldchange	65		42		333		96	
>1.5, <−1.5	27↑	38↓	2↑	40↓	175↑	158↓	21↑	75↓
	↓		↓		↓		↓	
Avg. sign.	63		35		333		96	
>50	37↑	26↓	2↑	33↓	175↑	158↓	21↑	75↓

Multiple testing corrections using False Discovery Rate was applied. A significant up-regulation was defined as a foldchange >1.5 and a significant down-regulation was defined as foldchange <−1.5. A minimum signal intensity value of 50 was utilized. Cm, cardiomyocytes; Fb, fibroblasts; sup. supernatant after ultracentrifugation, Avg. sign., average signal; FDR, False Discovery Rate; ↑, up-regulated; ↓, down-regulated.

Gene expression in cardiomyocytes treated with n-HA and o-HA was analyzed to determine if HA mediate intracellular signals leading to changes in gene transcription in cardiomyocytes. The addition of n-HA and o-HA to Claycomb media before incubation for 24 h, affected cardiomyocytes gene expression. When n-HA was added to Claycomb media 35 differentially expressed genes were detected, and 63 genes were detected with o-HA added (Table 2) (Table S3, S4). Data and information is available through GEO (accession number GSE21676).

## Discussion

In this study HA synthesis was investigated in two major cell types in heart tissue, cultured cardiomyocytes and fibroblasts to better understand the function of HA in the heart.

While fibroblasts are recognized as the main source of HA in connective tissues including in the heart, in this study the ability of cardiomyocytes to synthesize HA is reported. Cardiomyocytes cultured without growth factors or stimulated with FGF2 or TGFB2 synthesized no detectable levels of HA. However, the higher concentration of PDGF-BB (100 ng/ml) activated synthesis of HA in cardiomyocytes. Thus, cardiomyocytes must express HAS and indeed, confirmed by real-time PCR findings showing both *Has2* and *Has3* transcription. The absence of HA synthesis at the lower PDGF-BB concentration may indicate a threshold above which synthesis is induced in cardiomyocytes. It is possible that while fibroblasts are the primary source of HA in the myocardium, cardiomyocytes could be a secondary producer of HA that initiate their synthesis when PDGF-BB levels are high. The range in hydrodynamic diameter of the HA synthesized coincided to some extent the sizes of HA seen in fibroblasts and co-cultured cell controls. Thus, cardiomyocytes might produce HA of similar size to fibroblasts when an acute need for high amounts of HA arises to support HA synthesis derived from fibroblasts.

Adding HA to cardiomyocyte cultures showed that both oligo HA and native HA influence transcription of cardiomyocyte genes. Hence, cardiomyocytes were shown here to both produce and be affected by HA in their environment.

A pattern of decreased concentration and size of HA was observed with increased concentration of FGF2 or PDGF-BB in media from both fibroblasts and co-cultured cells (80% cardiomyocytes and 20% fibroblasts). This may be explained by hyaluronidase expression induced by FGF2 and PDGF-BB. Induction of hyaluronidases by PDGF-BB has been shown in human dermal fibroblasts [6]. The exception from this pattern was co-cultured cells stimulated with PDGF-BB at the higher concentration. Most likely, the cardiomyocytes contributed to the observed higher HA concentration, stimulated by PDGF-BB at the higher concentration. In co-cultures, containing only 20% fibroblasts and without addition of growth factors, HA was synthesized in equal amounts compared to fibroblast controls. It is unlikely that cardiomyocytes contributed to that HA synthesis, since cardiomyocytes alone did not synthesize HA at all, indicating a synergistic effect between the cell types. Furthermore, sizes of HA were larger in media from co-cultures than from cardiomyocytes alone, indicating an additional control of the synthesis derived from the interaction of the two different cell types.

In co-cultured cells, stimulated by FGF2 and PDGF-BB, this synergistic effect may be masked by increased hyaluronidase activity which could explain the low levels of HA seen in co-cultured cells compared to stimulated fibroblasts alone.

The synergistic effect on HA synthesis seen in co-cultured cells affecting seems to be caused by intercellular signaling between cardiomyocytes and fibroblasts. When culturing fibroblasts with media previously incubated with cardiomyocytes, an increase in HA concentration was observed. However, fibroblasts incubated with unused cardiomyocyte media did not increase their HA synthesis. Ultracentrifugation of the HA synthesis inducing media revealed that the HA inducing factor(s) was (were) to be found in the supernatant, thus most likely a soluble molecule. Microarray gene expression analysis of fibroblasts incubated with HA inducing media revealed a number of differently expressed genes, of which *Tgfb2* was one. The *Tgfb2* gene was 3-fold up-regulated both in fibroblasts incubated with total media from cardiomyocytes as well as cardiomyocyte media supernatant, showing a consistent effect on the regulation of the *Tgfb2* gene. Exogenously added TGFB2 to fibroblast media revealed TGFB2 to be a potent inducer of HA synthesis in the fibroblasts, where a 6 times higher HA concentration compared to controls was observed. *Tgfb2* has previously been shown to be the highest up-regulated growth factor gene in hypertrophic cardiac tissue, both in early and late state of the hypertrophic process, where both expression of *Has1* and *Has2* as well as increased HA synthesis have been observed [31]. In a recent publication it was shown in the developing heart that TGFB2 modulates *Has2* expression and HA production with subsequent induction of epithelial to mesenchymal transformation [32]. In the co-cultured cells with only 20% fibroblasts, TGFB2 induced a doubling of HA concentration compared to TGFB2 treated fibroblasts alone. This may be caused by a cumulative effect of the observed intercellular signaling from cardiomyocytes to fibroblasts, with subsequent up-regulation the *Tgfb2* gene together with the exogenously added TGFB2.

Cardiomyocyte controls expressed several genes coding for growth factors which could be candidate genes for the unknown factor secreted by cardiomyocytes, which might include connective tissue growth factor (*Ccn2*/*Ctgf*), insulin-like growth factor 1 and 2 (*Igf-1, 2*) and *Tgfb1*. TGFB1 has been shown to up-regulate the *Tgfb2* gene in a pancreatic cancer cell line [33]. However, it has also been reported that CCN2 acts as a cofactor to TGFB1, which supports the possibility that the HA synthesis inducer can be a combination of two factors [34].

The addition of growth factors in cell media consistently affected the HA size, demonstrating that growth factors not only influence the amount of synthesized HA also induced a change in HA size. In most cases, the cells produced HA in smaller size when stimulated with growth factors. The predominant synthase gene expressed was *Has2*, suggesting that HA size was not controlled by shift in HAS expression.

If the reduced size is caused by hyaluronidase activity, the short o-HA fragment most likely also mediates intracellular signaling, as demonstrated by the change in gene expression in cardiomyocytes by adding o-HA.

Both *Has1* and *Has2* have been detected in myocardial tissue from growing rat hearts [24]. In cultured cardiomyocytes and fibroblasts, only *Has2* (and not *Has1*) was detected both in controls and all growth factor treated combinations. In PDGF-BB treated cardiomyocytes and TGFB2 treated fibroblasts *Has3* was also expressed. One reason for *Has1* not being expressed could be the absence of vascular cells in the cell cultures. This is in concordance with a work by Li et. al. where PDGF-BB induced *Has* expression in human dermal fibroblasts showed a high expression of *Has2* with *Has1* and *Has3* also expressed but at low levels [6].

The ability of cardiomyocytes to send out a request to fibroblasts to synthesize HA reveals that cardiomyocytes need HA. When fibroblasts respond with HA synthesis, the cardiomyocytes detect the HA signal, and after signal transduction, a changed gene transcription in the cardiomyocytes is achieved.

In cardiac tissue, this signaling crosstalk can effectively be carried out. However, when the different cell types are cultured alone, the important crosstalk between cells is lost. If the cardiomyocyte fails to detect HA in its proximity, it will not undergo the following gene transcriptional changes. Thus, it is evident that cardiomyocytes collaborate with fibroblasts to optimize growth, where HA is a part of the cardiomyocyte extracellular matrix.

The discovery that cardiomyocytes under certain circumstances can synthesize HA themselves increases our recognition of further possible regulatory activities of cardiomyocytes.

## Supporting Information

Table S1 Differentially expressed genes in fibroblasts cultured in media previously incubated with cardiomyocytes.
(0.49 MB DOC)

Table S2 Differentially expressed genes in fibroblasts cultured in media supernatant from ultrcentrifugation, previously incubated with cardiomyocytes.
(0.16 MB DOC)

Table S3 Differentially expressed genes in cardiomyocytes cultured in medium with added native-HA.
(0.07 MB DOC)

Table S4 Differentially expressed genes in cardiomyocytes cultured in medium with added oligo-HA.
(0.11 MB DOC)
