# Supplementary material for: Growth Factor PDGF-BB Stimulates Cultured Cardiomyocytes to Synthesize the Extracellular Matrix Component Hyaluronan
Source: PLoS One. 2010 Dec 21;5(12):e14393. doi: 10.1371/journal.pone.0014393 (PMC3006157; doi:10.1371/journal.pone.0014393)
Supplement: Table S1 — Differentially expressed genes in fibroblasts cultured in media previously incubated with cardiomyocytes. (0.49 MB DOC) [file pone.0014393.s001.doc]

| Table S1. Differentially expressed genes in fibroblasts cultured in media previously incubated with cardiomyocytes. | | | | | | | | |
| --- | --- | --- | --- | --- | --- | --- | --- | --- |
| ENTREZ GENE ID | SYMBOL | DEFINITION | Foldchange | Diff *P*-value | Treated cells average signal | Control average signal | Treated cells detection *P*-value | Control detection *P*-value |
| 19074 | Prg2 | Mus musculus proteoglycan 2, bone marrow (Prg2), mRNA. | 22,58126 | 8,5921E-35 | 612,2823 | 27,11462 | 0 | 0,01253133 |
| 18792 | Plau | Mus musculus plasminogen activator, urokinase (Plau), mRNA. | 20,27095 | 8,5921E-35 | 1528,203 | 75,38884 | 0 | 0 |
| 56096 | Plac1 | Mus musculus placental specific protein 1 (Plac1), mRNA. | 15,01346 | 8,5921E-35 | 414,574 | 27,61348 | 0 | 0,00877193 |
| 231507 | Plac8 | Mus musculus placenta-specific 8 (Plac8), mRNA. | 13,2329 | 2,5311E-07 | 350,1186 | 26,45818 | 0 | 0,01253133 |
| 232441 | Rerg | Mus musculus RAS-like, estrogen-regulated, growth-inhibitor (Rerg), mRNA. | 11,4335 | 1,4324E-07 | 228,175 | 19,95672 | 0 | 0,02882206 |
| 16323 | Inhba | Mus musculus inhibin beta-A (Inhba), mRNA. | 10,95979 | 1,902E-12 | 225,5283 | 20,57778 | 0 | 0,02255639 |
| 17067 | Ly6c1 | Mus musculus lymphocyte antigen 6 complex, locus C1 (Ly6c1), mRNA. | 10,7324 | 7,4835E-11 | 191,2685 | 17,82159 | 0 | 0,04511278 |
| 20292 | Ccl11 | Mus musculus small chemokine (C-C motif) ligand 11 (Ccl11), mRNA. | 9,905057 | 1,9295E-22 | 417,9742 | 42,19806 | 0 | 0,000153894 |
| 20308 | Ccl9 | Mus musculus chemokine (C-C motif) ligand 9 (Ccl9), mRNA. | 9,623677 | 1,902E-12 | 244,4885 | 25,40489 | 0 | 0,01629073 |
| 14114 | Fbln1 | Mus musculus fibulin 1 (Fbln1), mRNA. | 9,019203 | 3,0657E-12 | 149,2429 | 16,54723 | 0,000999871 | 0,008679383 |
| 240913 | Adamts4 | Mus musculus a disintegrin-like and metallopeptidase (reprolysin type) with thrombospondin type 1 motif, 4, mRNA | 8,738959 | 2,4093E-05 | 399,1433 | 45,674 | 0 | 0 |
| 223272 | Itgbl1 | Mus musculus integrin, beta-like 1 (Itgbl1), mRNA. | 8,245728 | 8,5921E-35 | 715,3473 | 86,75369 | 0 | 0 |
| 116847 | Prelp | Mus musculus proline arginine-rich end leucine-rich repeat (Prelp), mRNA. | 7,767286 | 7,6078E-13 | 2101,285 | 270,5301 | 0 | 0 |
| 11826 | Aqp1 | Mus musculus aquaporin 1 (Aqp1), mRNA. | 7,725135 | 8,1774E-26 | 1972,474 | 255,3319 | 0 | 0 |
| 21683 | Tecta | Mus musculus tectorin alpha (Tecta), mRNA. | 7,301977 | 1,1755E-07 | 206,2283 | 28,2428 | 0 | 0,00877193 |
| 100048554 | LOC100048554 | PREDICTED: Mus musculus similar to monocyte chemoattractant protein-2 (MCP-2) (LOC100048554), mRNA. | 6,9247 | 0,00287354 | 737,5638 | 106,512 | 0 | 0 |
| 235283 | Gramd1b | Mus musculus GRAM domain containing 1B (Gramd1b), mRNA. | 6,834915 | 6,389E-07 | 116,9837 | 17,11561 | 0 | 0,03508772 |
| 50530 | Mfap5 | Mus musculus microfibrillar associated protein 5 (Mfap5), mRNA. | 6,756049 | 8,5921E-35 | 1108,712 | 164,1066 | 0 | 0 |
| 20296 | Ccl2 | Mus musculus chemokine (C-C motif) ligand 2 (Ccl2), mRNA. | 6,681957 | 1,902E-12 | 360,0652 | 53,88618 | 0 | 0 |
| 30959 | Ddx25 | Mus musculus DEAD (Asp-Glu-Ala-Asp) box polypeptide 25 (Ddx25), mRNA. | 5,680216 | 1,3504E-10 | 437,0157 | 76,93646 | 0 | 0 |
| 72324 | Plxdc1 | Mus musculus plexin domain containing 1 (Plxdc1), mRNA. | 5,544508 | 1,0574E-06 | 328,2634 | 59,20515 | 0 | 0 |
| 22341 | Vegfc | Mus musculus vascular endothelial growth factor C (Vegfc), mRNA. | 5,453966 | 0,00475776 | 125,5737 | 23,02428 | 0 | 0,01754386 |
| 654450 | Klra33 | Mus musculus killer cell lectin-like receptor subfamily A member 33 (Klra33), mRNA. | 5,401996 | 0,00972608 | 160,8191 | 29,77031 | 0 | 0,00877193 |
| 240725 | Sulf1 | Mus musculus sulfatase 1 (Sulf1), mRNA. | 5,030806 | 4,7909E-08 | 309,649 | 61,55057 | 0 | 0 |
| 83436 | Plekha2 | Mus musculus pleckstrin homology domain-containing, family A (phosphoinositide binding specific) member 2  (Plekha2), mRNA. | 4,84756 | 2,1022E-12 | 1008,948 | 208,1352 | 0 | 0 |
| 16773 | Lama2 | Mus musculus laminin, alpha 2 (Lama2), mRNA. | 4,792711 | 0,00253176 | 113,8631 | 23,75756 | 0 | 0,01754386 |
| 68728 | Trp53inp2 | Mus musculus transformation related protein 53 inducible nuclear protein 2 (Trp53inp2), mRNA. | 4,788995 | 4,7967E-08 | 206,177 | 43,05224 | 0 | 0 |
| 213945 | Col28a1 | Mus musculus collagen, type XXVIII, alpha 1 (Col28a1), mRNA. | 4,3799 | 1,4105E-06 | 156,6629 | 35,76861 | 0 | 0,005012531 |
| 102644 | Oaf | Mus musculus OAF homolog (Drosophila) (Oaf), mRNA. | 4,296156 | 0,00213267 | 79,48403 | 18,5012 | 0 | 0,02882206 |
| 54409 | Ramp2 | Mus musculus receptor (calcitonin) activity modifying protein 2 (Ramp2), mRNA. | 4,255418 | 7,5917E-08 | 206,3794 | 48,49804 | 0 | 0 |
| 72774 | Neil1 | Mus musculus nei endonuclease VIII-like 1 (E. coli) (Neil1), mRNA. | 4,170623 | 0,000509 | 89,65711 | 21,49729 | 0 | 0,01879699 |
| 12362 | Casp1 | Mus musculus caspase 1 (Casp1), mRNA. | 4,161228 | 0,02863997 | 92,48812 | 22,22616 | 0 | 0,01754386 |
| 81840 | Sorcs2 | Mus musculus sortilin-related VPS10 domain containing receptor 2 (Sorcs2), mRNA. | 4,043403 | 2,5808E-05 | 120,5193 | 29,8064 | 0 | 0,006265664 |
| 380969 | C230021P08Rik | Mus musculus Riken cDNA C230021P08 gene (C230021P08Rik), mRNA. | 3,853125 | 0,00827132 | 75,46383 | 19,5851 | 0 | 0,002641315 |
| 26364 | Cd97 | Mus musculus CD97 antigen (Cd97), mRNA. | 3,852793 | 0,00025582 | 317,512 | 82,41087 | 0 | 2,24958E-06 |
| 27528 | D0H4S114 | Mus musculus DNA segment, human D4S114 (D0H4S114), mRNA. | 3,839749 | 6,4169E-09 | 1576,942 | 410,6888 | 0 | 0 |
| 217066 | BC099439 | Mus musculus cDNA sequence BC099439 (BC099439), mRNA. | 3,831192 | 0,00036893 | 438,6235 | 114,4875 | 0 | 0 |
| 20356 | Sema5a | Mus musculus sema domain, seven thrombospondin repeats (type 1 and type 1-like), transmembrane domain (TM) and  short cytoplasmic domain, (semaphorin) 5A (Sema5a), mRNA. | 3,822105 | 5,2542E-10 | 1314,464 | 343,911 | 0 | 0 |
| 18769 | Pkig | Mus musculus protein kinase inhibitor, gamma (Pkig), transcript variant 3, mRNA. | 3,737151 | 0,00012691 | 172,4838 | 46,15382 | 0 | 0,01105217 |
| 114564 | Csprs | Mus musculus component of Sp100-rs (Csprs), mRNA. | 3,706254 | 0,01003963 | 64,96263 | 17,52784 | 0 | 0,03508772 |
| 56213 | Htra1 | Mus musculus HtrA serine peptidase 1 (Htra1), mRNA. | 3,694879 | 3,2323E-05 | 116,5741 | 31,55018 | 0 | 0,01452252 |
| 20377 | Sfrp1 | Mus musculus secreted frizzled-related protein 1, mRNA | 3,662801 | 0,00073582 | 464,1383 | 126,7168 | 0 | 0 |
| 83397 | Akap12 | Mus musculus A kinase (PRKA) anchor protein (gravin) 12 (Akap12), mRNA. | 3,598114 | 3,4128E-05 | 1711,102 | 475,5552 | 0 | 0 |
| 56429 | Dpt | Mus musculus dermatopontin (Dpt), mRNA. | 3,572629 | 0,0112699 | 1128,431 | 315,8545 | 0 | 0 |
| 319186 | Hist1h2bm | Mus musculus histone cluster 1, H2bm (Hist1h2bm), mRNA. | 3,561818 | 0,04753023 | 60,36257 | 16,94712 | 0 | 0,03884712 |
| 26432 | Plod2 | Mus musculus procollagen lysine, 2-oxoglutarate 5-dioxygenase 2 (Plod2), mRNA. | 3,560893 | 1,6063E-07 | 288,4041 | 80,9921 | 0 | 0 |
| 110454 | Ly6a | Mus musculus lymphocyte antigen 6 complex, locus A (Ly6a), mRNA. | 3,376023 | 9,3185E-10 | 3658,236 | 1083,593 | 0 | 0 |
| 11475 | Acta2 | Mus musculus actin, alpha 2, smooth muscle, aorta (Acta2), mRNA. | 3,372275 | 1,0253E-05 | 2091,548 | 620,2186 | 0,00026605 | 0,00026605 |
| 207521 | Dtx4 | Mus musculus deltex 4 homolog (Drosophila) (Dtx4), mRNA. XM_001000490 | 3,341388 | 5,2605E-06 | 612,0862 | 183,1832 | 0 | 0 |
| 21808 | Tgfb2 | Mus musculus transforming growth factor, beta 2 (Tgfb2), mRNA. | 3,273472 | 3,7486E-08 | 525,5394 | 160,5449 | 0 | 0 |
| 68659 | 1110032E23Rik | Mus musculus RIKEN cDNA 1110032E23 gene (1110032E23Rik), mRNA. | 3,268121 | 4,9182E-08 | 3617,637 | 1106,947 | 0 | 0 |
| 100038894 | LOC100038894 | PREDICTED: Mus musculus hypothetical protein LOC100038894 (LOC100038894), mRNA. | 3,202958 | 2,7069E-05 | 556,94 | 173,883 | 0 | 0 |
| 244418 | D8Ertd82e | Mus musculus DNA segment, Chr 8, ERATO Doi 82, expressed (D8Ertd82e), mRNA. | 3,199466 | 0,04753023 | 94,16129 | 29,43031 | 0 | 0,006265664 |
| 106042 | Prickle1 | Mus musculus prickle like 1 (Drosophila) (Prickle1), mRNA. | 3,181022 | 6,0061E-06 | 615,0174 | 193,3396 | 0 | 0 |
| 81910 | Rrbp1 | Mus musculus ribosome binding protein 1 (Rrbp1), transcript variant 1, mRNA. | 3,146968 | 0,00776037 | 420,3914 | 133,5862 | 0 | 0 |
| 11486 | Ada | Mus musculus adenosine deaminase (Ada), mRNA. | 3,108401 | 0,03124758 | 52,62014 | 16,92836 | 0,000754549 | 0,01886373 |
| 171504 | Apob48r | Mus musculus apolipoprotein B48 receptor (Apob48r), mRNA. | 3,043651 | 0,00234455 | 117,7647 | 38,69194 | 3,14068E-06 | 0,004416586 |
| 235050 | Zfp810 | Mus musculus zinc finger protein 810 (Zfp810), mRNA. | 3,007963 | 0,01307665 | 91,6348 | 30,46407 | 0 | 0,006265664 |
| 16477 | Junb | Mus musculus Jun-B oncogene (Junb), mRNA. | 3,006413 | 5,2824E-07 | 431,5528 | 143,5441 | 0 | 0 |
| 66355 | Gmpr | Mus musculus guanosine monophosphate reductase (Gmpr), mRNA. | 3,004243 | 0,02189367 | 206,9543 | 68,88734 | 0 | 0 |
| 381413 | Gpr176 | Mus musculus G protein-coupled receptor 176 (Gpr176), mRNA. | 2,989864 | 6,4168E-06 | 237,7042 | 79,50334 | 0 | 0 |
| 17022 | Lum | Mus musculus lumican (Lum), mRNA. | 2,970245 | 1,0069E-07 | 11966,01 | 4028,626 | 0 | 0 |
| 246256 | Fcgr4 | Mus musculus Fc receptor, IgG, low affinity IV (Fcgr4), mRNA. | 2,949633 | 0,01499461 | 158,0327 | 53,57708 | 0 | 0 |
| 13642 | Efnb2 | Mus musculus ephrin B2 (Efnb2), mRNA. | 2,925292 | 0,02694601 | 71,08762 | 24,30103 | 0 | 0,01754386 |
| 66873 | 1200009O22Rik | Mus musculus RIKEN cDNA 1200009O22 gene (1200009O22Rik), mRNA. | 2,862031 | 6,7032E-07 | 506,7736 | 177,0678 | 0 | 0 |
| 26427 | Creb3l1 | Mus musculus cAMP responsive element binding protein 3-like 1 (Creb3l1), mRNA. | 2,827765 | 0,00148 | 116,9111 | 41,34399 | 0 | 0,002506266 |
| 56312 | Nupr1 | Mus musculus nuclear protein 1 (Nupr1), mRNA. | 2,773387 | 1,5734E-07 | 2354,542 | 848,9769 | 0 | 0 |
| 70399 | 2310058J06Rik | Mus musculus RIKEN cDNA 2310058J06 gene (2310058J06Rik), mRNA | 2,748102 | 0,03854831 | 114,1879 | 41,55157 | 0 | 0,001253133 |
| 12051 | Bcl3 | Mus musculus B-cell leukemia/lymphoma 3 (Bcl3), mRNA. | 2,685782 | 0,00961047 | 100,1885 | 37,30328 | 0 | 0,003759399 |
| 320078 | Olfml2b | Mus musculus olfactomedin-like 2B (Olfml2b), mRNA. | 2,68058 | 1,2336E-05 | 592,6187 | 221,0786 | 0 | 0 |
| 13038 | Ctsk | Mus musculus cathepsin K (Ctsk), mRNA. | 2,657532 | 5,6261E-07 | 1663,101 | 625,8066 | 0 | 0 |
| 17395 | Mmp9 | Mus musculus matrix metallopeptidase 9 (Mmp9), mRNA. | 2,627236 | 0,00093908 | 201,9115 | 76,8532 | 0 | 0 |
| 677289 | ENSMUSG00000043795 | PREDICTED: Mus musculus predicted gene, ENSMUSG00000043795 (ENSMUSG00000043795), mRNA. | 2,603648 | 7,3508E-07 | 2326,626 | 893,6023 | 0 | 0 |
| 16010 | Igfbp4 | Mus musculus insulin-like growth factor binding protein 4, mRNA | 2,590463 | 1,5907E-05 | 440,894 | 170,1989 | 0 | 0 |
| 11754 | Aoc3 | Mus musculus amine oxidase, copper containing 3 (Aoc3), mRNA. | 2,57769 | 0,04822861 | 299,3631 | 116,1362 | 0 | 0 |
| 18035 | Nfkbia | Mus musculus nuclear factor of kappa light polypeptide gene enhancer in B-cells inhibitor, alpha (Nfkbia), mRNA. | 2,570605 | 5,789E-05 | 545,4923 | 212,2039 | 0 | 0 |
| 57257 | Vav3 | Mus musculus vav 3 oncogene (Vav3), transcript variant 1, mRNA. | 2,565213 | 0,01921681 | 69,89083 | 27,24563 | 0,001063225 | 0,002214244 |
| 18805 | Pld1 | Mus musculus phospholipase D1, mRNA | 2,550023 | 0,00040228 | 314,4261 | 123,3032 | 0 | 0 |
| 17069 | Ly6e | Mus musculus lymphocyte antigen 6 complex, locus E (Ly6e), mRNA. | 2,528955 | 5,75E-05 | 1057,757 | 418,2584 | 0 | 0 |
| 76491 | Abhd14b | Mus musculus abhydrolase domain containing 14b (Abhd14b), mRNA. | 2,514145 | 0,00028451 | 255,9025 | 101,7851 | 0 | 0 |
| 107589 | Mylk | Mus musculus myosin, light polypeptide kinase (Mylk), mRNA. | 2,492754 | 0,00767405 | 182,5016 | 73,21285 | 0 | 1,96293E-05 |
| 103677 | Smg6 | Mus musculus Smg-6 homolog, nonsense mediated mRNA decay factor (C. elegans) (Smg6), mRNA. | 2,491242 | 0,00010325 | 706,7785 | 283,7053 | 0 | 0 |
| 319183 | Hist1h2bj | Mus musculus histone cluster 1, H2bj (Hist1h2bj), mRNA. | 2,490614 | 0,00150469 | 146,245 | 58,71846 | 0 | 0 |
| 76781 | Mettl4 | Mus musculus methyltransferase like 4 (Mettl4), mRNA. | 2,487402 | 0,03078957 | 85,47239 | 34,36211 | 0 | 0,005012531 |
| 22359 | Vldlr | Mus musculus very low density lipoprotein receptor (Vldlr), mRNA. | 2,461746 | 0,0028007 | 398,9984 | 162,0794 | 0,001751444 | 0,01253529 |
| 214791 | Sertad4 | Mus musculus SERTA domain containing 4 (Sertad4), mRNA. | 2,458265 | 0,00676083 | 842,772 | 342,8321 | 0 | 0,001133787 |
| 19703 | Renbp | Mus musculus renin binding protein (Renbp), mRNA. | 2,454924 | 1,0855E-05 | 672,7604 | 274,0453 | 0 | 0 |
| 100046120 | LOC100046120 | PREDICTED: Mus musculus similar to clusterin (LOC100046120), mRNA. | 2,446465 | 6,4168E-06 | 1060,859 | 433,6295 | 0 | 0 |
| 17289 | Mertk | Mus musculus c-mer proto-oncogene tyrosine kinase (Mertk), mRNA. | 2,436923 | 0,01835551 | 442,4023 | 181,5413 | 0 | 0 |
| 399558 | Flrt2 | Mus musculus fibronectin leucine rich transmembrane protein 2 (Flrt2), mRNA. | 2,435177 | 0,0325069 | 83,65302 | 34,35193 | 0 | 0,005012531 |
| 71660 | Rarres2 | Mus musculus retinoic acid receptor responder (tazarotene induced) 2 (Rarres2), mRNA. | 2,412296 | 0,00181442 | 153,5105 | 63,63668 | 0 | 0 |
| 14118 | Fbn1 | Mus musculus fibrillin 1, mRNA | 2,404605 | 0,00085198 | 511,6298 | 212,7708 | 0 | 0 |
| 66569 | Gdpd1 | Mus musculus glycerophosphodiester phosphodiesterase domain containing 1 (Gdpd1), mRNA. | 2,400852 | 3,2803E-05 | 489,4975 | 203,8849 | 0 | 0 |
| 20482 | Skil | Mus musculus SKI-like (Skil), transcript variant 1, mRNA. | 2,398642 | 0,00455918 | 170,5148 | 71,08803 | 0,007086168 | 0,003116343 |
| 74202 | Fblim1 | Mus musculus filamin binding LIM protein 1 (Fblim1), mRNA. | 2,397792 | 0,00030902 | 387,4172 | 161,5725 | 0 | 0 |
| 75608 | Chmp4b | Mus musculus chromatin modifying protein 4B (Chmp4b), mRNA. | 2,365099 | 0,00097667 | 782,3943 | 330,8082 | 0 | 0 |
| 100045780 | LOC100045780 | PREDICTED: Mus musculus similar to metalloprotease-disintegrin meltrin beta (LOC100045780), mRNA. | 2,346621 | 2,4039E-05 | 798,1349 | 340,121 | 0 | 0 |
| 319182 | Hist1h2bh | Mus musculus histone cluster 1, H2bh (Hist1h2bh), mRNA. | 2,335136 | 0,0279268 | 119,9802 | 51,38041 | 0 | 0 |
| 12825 | Col3a1 | Mus musculus collagen, type III, alpha 1 (Col3a1), mRNA. | 2,329273 | 0,00430596 | 1693,593 | 727,0906 | 0 | 0 |
| 213019 | Pdlim2 | Mus musculus PDZ and LIM domain 2 (Pdlim2), mRNA. | 2,326739 | 0,00065321 | 350,0765 | 150,458 | 0,00026605 | 0,00026605 |
| 100046393 | LOC100046393 | PREDICTED: Mus musculus similar to Protein phosphatase 2, regulatory subunit B (B56), alpha (LOC100046393), mRNA. | 2,310818 | 0,00012134 | 421,2119 | 182,2783 | 0 | 0 |
| 22339 | Vegfa | Mus musculus vascular endothelial growth factor A (Vegfa), transcript variant 1, mRNA. | 2,304693 | 0,00194802 | 455,8318 | 197,7842 | 0,0463824 | 0,004042742 |
| 52552 | Parp8 | Mus musculus poly (ADP-ribose) polymerase family, member 8 (Parp8), mRNA. | 2,304242 | 0,04385246 | 115,4676 | 50,11089 | 0 | 0 |
| 21899 | Tlr6 | Mus musculus toll-like receptor 6 (Tlr6), mRNA. | 2,291688 | 0,02612559 | 98,18067 | 42,84208 | 0 | 0,001253133 |
| 20250 | Scd2 | Mus musculus stearoyl-Coenzyme A desaturase 2 (Scd2), mRNA. | 2,273619 | 5,6965E-05 | 711,9163 | 313,1203 | 0 | 0 |
| 19369 | Raet1b | Mus musculus retinoic acid early transcript beta (Raet1b), mRNA. | 2,232045 | 0,00015259 | 524,5988 | 235,0306 | 0 | 0 |
| 12125 | Bcl2l11 | Mus musculus BCL2-like 11 (apoptosis facilitator), mRNA | 2,230468 | 0,01268191 | 177,9027 | 79,76027 | 0,004898961 | 0,0149029 |
| 13654 | Egr2 | Mus musculus early growth response 2, mRNA | 2,227151 | 0,00102848 | 893,7955 | 401,318 | 0 | 0 |
| 18438 | P2rx4 | Mus musculus purinergic receptor P2X, ligand-gated ion channel 4 (P2rx4), mRNA. | 2,222693 | 0,01905408 | 197,5892 | 88,89629 | 0 | 7,06654E-06 |
| 18709 | Pik3r2 | Mus musculus phosphatidylinositol 3-kinase, regulatory subunit, polypeptide 2 (p85 beta) (Pik3r2), mRNA. | 2,206926 | 0,00924981 | 281,5 | 127,553 | 0 | 0 |
| 19223 | Ptgis | Mus musculus prostaglandin I2 (prostacyclin) synthase (Ptgis), mRNA. | 2,204516 | 5,3901E-05 | 1729,948 | 784,7291 | 0 | 0 |
| 674706 | LOC674706 | PREDICTED: Mus musculus similar to Zinc finger protein 341 (LOC674706), mRNA. | 2,201704 | 0,03814837 | 251,8175 | 114,3739 | 0 | 0 |
| 100201 | 9630015D15Rik | Mus musculus RIKEN cDNA 9630015D15Rik gene (9630015D15Rik), mRNA | 2,187667 | 0,0487023 | 213,2646 | 97,48497 | 0 | 0 |
| 20306 | Ccl7 | Mus musculus chemokine (C-C motif) ligand 7 (Ccl7), mRNA. | 2,183678 | 5,0573E-05 | 3876,148 | 1775,054 | 0 | 0 |
| 12827 | Col4a2 | Mus musculus collagen, type IV, alpha 2 (Col4a2), mRNA. | 2,177052 | 0,04171725 | 1801,31 | 827,4077 | 0 | 0 |
| 11541 | Adora2b | Mus musculus adenosine A2b receptor (Adora2b), mRNA. | 2,169175 | 8,6439E-05 | 1435,167 | 661,6188 | 0 | 0 |
| 66270 | Fam134b | Mus musculus family with sequence similarity 134, member B (Fam134b), transcript variant 2, mRNA. | 2,15672 | 0,00081643 | 324,6034 | 150,5079 | 0 | 0 |
| 67732 | 4833421E05Rik | Mus musculusRIKEN cDNA 4833421E05Rik gene (4833421E05Rik), mRNA | 2,152151 | 0,00220684 | 276,8855 | 128,6552 | 0 | 0 |
| 319180 | Hist1h2bf | Mus musculus histone cluster 1, H2bf (Hist1h2bf), mRNA. | 2,150014 | 0,00495118 | 207,9991 | 96,74311 | 0 | 0 |
| 19159 | Pscd3 | Mus musculus pleckstrin homology, Sec7 and coiled-coil domains 3 (Pscd3), mRNA. | 2,138001 | 0,00057819 | 3027,934 | 1416,245 | 0 | 0 |
| 68026 | 2810417H13Rik | Mus musculus RIKEN cDNA 2810417H13 gene (2810417H13Rik), mRNA. | 2,108187 | 0,00572796 | 186,3432 | 88,39026 | 0 | 0 |
| 14595 | B4galt1 | Mus musculus UDP-Gal:betaGlcNAc beta 1,4- galactosyltransferase, polypeptide 1 (B4galt1), mRNA. | 2,095371 | 0,00048832 | 572,5867 | 273,2626 | 0 | 0 |
| 12389 | Cav1 | Mus musculus caveolin 1, caveolae protein (Cav1), mRNA. | 2,077024 | 0,00016592 | 4395,689 | 2116,34 | 0 | 0 |
| 74194 | Rnd3 | Mus musculus Rho family GTPase 3 (Rnd3), mRNA. | 2,061599 | 0,02329596 | 133,6412 | 64,82402 | 0 | 0 |
| 23966 | Odz4 | Mus musculus odd Oz/ten-m homolog 4 (Drosophila) (Odz4), mRNA. | 2,059995 | 0,00270378 | 455,0434 | 220,8954 | 0 | 0 |
| 97064 | Wwtr1 | Mus musculus WW domain containing transcription regulator 1 (Wwtr1), mRNA. | 2,059225 | 0,02419168 | 141,1689 | 68,55439 | 0 | 0 |
| 67732 | Iah1 | Mus musculus isoamyl acetate-hydrolyzing esterase 1 homolog (S. cerevisiae) (Iah1), mRNA. | 2,05689 | 0,00081229 | 526,4068 | 255,9237 | 0 | 0 |
| 66199 | Commd4 | Mus musculus COMM domain containing 4 (Commd4), mRNA. | 2,038635 | 0,00040775 | 1343,524 | 659,0312 | 0 | 0 |
| 58194 | Sh3kbp1 | Mus musculus SH3-domain kinase binding protein 1 (Sh3kbp1), mRNA. | 2,036786 | 0,00114209 | 1526,426 | 749,429 | 0,000270375 | 0,005055231 |
| 21928 | Tnfaip2 | Mus musculus tumor necrosis factor, alpha-induced protein 2 (Tnfaip2), mRNA. | 2,032161 | 0,00104065 | 497,873 | 244,9969 | 0 | 0 |
| 71910 | Ppapdc1b | Mus musculus phosphatidic acid phosphatase type 2 domain containing 1B (Ppapdc1b), mRNA. | 2,021832 | 0,00194815 | 372,7344 | 184,3548 | 0 | 0 |
| 54132 | Pdlim1 | Mus musculus PDZ and LIM domain 1 (elfin) (Pdlim1), mRNA. | 2,002846 | 0,00839078 | 500,8519 | 250,0701 | 0 | 0 |
| 18767 | Pkia | Mus musculus protein kinase inhibitor, alpha (Pkia), mRNA. | 1,961274 | 0,00364233 | 365,6949 | 186,4578 | 0 | 0 |
| 53893 | Nudt5 | Mus musculus nudix (nucleoside diphosphate linked moiety X)-type motif 5 (Nudt5), mRNA. | 1,95945 | 0,01877419 | 868,2913 | 443,1301 | 0 | 0 |
| 21973 | Top2a | Mus musculus topoisomerase (DNA) II alpha (Top2a), mRNA. | 1,956758 | 0,00287354 | 1502,679 | 767,9429 | 0 | 0 |
| 14230 | Fkbp10 | Mus musculus FK506 binding protein 10 (Fkbp10), mRNA. | 1,953883 | 0,02329596 | 723,3142 | 370,1932 | 0 | 0 |
| 15251 | Hif1a | Mus musculus hypoxia inducible factor 1, alpha subunit (Hif1a), mRNA. | 1,913941 | 0,01918017 | 331,8481 | 173,3847 | 0 | 0 |
| 67921 | 2510010F15Rik | Mus musculus RIKEN cDNA 2510010F15Rik gene (2510010F15Rik), mRNA | 1,884519 | 0,02672589 | 188,1991 | 99,86587 | 0 | 0 |
| 65972 | Ifi30 | Mus musculus interferon gamma inducible protein 30 (Ifi30), mRNA. | 1,875224 | 0,0294162 | 434,3593 | 231,6307 | 0 | 0 |
| 13617 | Ednra | Mus musculus endothelin receptor type A (Ednra), mRNA. | 1,86531 | 0,00238957 | 2423,178 | 1299,075 | 0 | 0 |
| 71934 | Car13 | Mus musculus carbonic anhydrase 13 (Car13), mRNA. | 1,862236 | 0,00297564 | 1407,141 | 755,6191 | 0 | 0 |
| 12826 | Col4a1 | Mus musculus procollagen, type IV, alpha 1 (Col4a1), mRNA. | 1,858182 | 0,00933935 | 3415,302 | 1837,98 | 0 | 0 |
| 230967 | BC046331 | Mus musculus cDNA sequence BC046331 (BC046331), mRNA. | 1,851034 | 0,02040065 | 241,0448 | 130,2217 | 0 | 0 |
| 12831 | Col5a1 | Mus musculus procollagen, type V, alpha 1 (Col5a1), mRNA. | 1,845939 | 0,04779637 | 7410,933 | 4014,723 | 0 | 0 |
| 72461 | Prcp | Mus musculus prolylcarboxypeptidase (angiotensinase C) (Prcp), mRNA. | 1,838235 | 0,00354063 | 1889,563 | 1027,923 | 0 | 0 |
| 12236 | Bub1b | Mus musculus budding uninhibited by benzimidazoles 1 homolog, beta (S. cerevisiae) (Bub1b), mRNA. | 1,835837 | 0,02009822 | 260,0083 | 141,6293 | 0 | 0 |
| 20249 | Scd1 | Mus musculus stearoyl-Coenzyme A desaturase 1 (Scd1), mRNA. | 1,795773 | 0,01331485 | 3310,711 | 1843,614 | 0 | 0 |
| 13653 | Egr1 | Mus musculus early growth response 1 (Egr1), mRNA. | 1,777761 | 0,0057989 | 8996,953 | 5060,833 | 0 | 0 |
| 13870 | Ercc1 | Mus musculus excision repair cross-complementing rodent repair deficiency, complementation group 1 (Ercc1), mRNA. | 1,777232 | 0,00986658 | 1527,655 | 859,5701 | 0 | 0 |
| 68214 | Gsto2 | Mus musculus glutathione S-transferase omega 2 (Gsto2), mRNA. | 1,771816 | 0,03774426 | 203,9236 | 115,093 | 0 | 0 |
| 12752 | Cln3 | Mus musculus ceroid lipofuscinosis, neuronal 3, juvenile (Batten, Spielmeyer-Vogt disease) (Cln3), mRNA. | 1,765039 | 0,03110137 | 286,3304 | 162,2233 | 0 | 0 |
| 19659 | Rbp1 | Mus musculus retinol binding protein 1, mRNA | 1,763769 | 0,00722322 | 4010,737 | 2273,958 | 0 | 0 |
| 59015 | Nup160 | Mus musculus nucleoporin 160 (Nup160), mRNA. | 1,75985 | 0,02338499 | 370,8496 | 210,728 | 0 | 0 |
| 21810 | Tgfbi | Mus musculus transforming growth factor, beta induced (Tgfbi), mRNA. | 1,755959 | 0,04273547 | 1201,345 | 684,1531 | 0 | 0 |
| 268396 | Sh3pxd2b | Mus musculus SH3 and PX domains 2B (Sh3pxd2b), mRNA. | 1,750805 | 0,04959452 | 734,9482 | 419,7772 | 0 | 0 |
| 69219 | Ddah1 | Mus musculus dimethylarginine dimethylaminohydrolase 1, mRNA | 1,75025 | 0,03369667 | 447,3975 | 255,6193 | 0 | 0 |
| 218454 | Lhfpl2 | Mus musculus lipoma HMGIC fusion partner-like 2 (Lhfpl2), mRNA. | 1,739378 | 0,00971389 | 3872,794 | 2226,539 | 0 | 0 |
| 216725 | Adamts2 | Mus musculus a disintegrin-like and metallopeptidase (reprolysin type) with thrombospondin type 1 motif, 2  (Adamts2), mRNA. | 1,729291 | 0,04905628 | 1773,595 | 1025,62 | 0 | 0 |
| 209773 | Dennd2a | Mus musculus DENN/MADD domain containing 2A (Dennd2a), mRNA. | 1,722463 | 0,01611616 | 1030,477 | 598,258 | 0 | 0 |
| 22433 | Xbp1 | Mus musculus X-box binding protein 1 (Xbp1), mRNA. | 1,720359 | 0,01260275 | 2767,82 | 1608,862 | 0 | 0 |
| 67468 | Mmd | Mus musculus monocyte to macrophage differentiation-associated (Mmd), mRNA. | 1,712979 | 0,01265014 | 5708,612 | 3332,565 | 0 | 0 |
| 53860 | Sept9 | Mus musculus septin 9 (Sept9), mRNA. | 1,709496 | 0,01801878 | 1123,458 | 657,1866 | 0 | 0 |
| 20440 | St6gal1 | Mus musculus beta galactoside alpha 2,6 sialyltransferase 1 (St6gal1), mRNA. | 1,705962 | 0,01476451 | 2863,504 | 1678,527 | 0 | 0 |
| 26943 | Serinc3 | Mus musculus serine incorporator 3 (Serinc3), mRNA. | 1,697942 | 0,02465067 | 728,1597 | 428,8485 | 0 | 0 |
| 110078 | Pygb | Mus musculus brain glycogen phosphorylase (Pygb), mRNA. | 1,697612 | 0,03258174 | 482,4356 | 284,1848 | 0 | 0 |
| 226791 | Lyplal1 | Mus musculus lysophospholipase-like 1 (Lyplal1), mRNA. | 1,696851 | 0,03584217 | 248,5644 | 146,4857 | 0 | 1,25627E-05 |
| 52502 | Carhsp1 | Mus musculus calcium regulated heat stable protein 1 (Carhsp1), mRNA. | 1,695289 | 0,02167416 | 1174,232 | 692,6441 | 0 | 0 |
| 320024 | Aadacl1 | Mus musculus arylacetamide deacetylase-like 1 (Aadacl1), mRNA. | 1,68932 | 0,04344358 | 1520,737 | 900,2064 | 0 | 0 |
| 18845 | Plxna2 | Mus musculus plexin A2 (Plxna2), mRNA. | 1,683841 | 0,02787751 | 760,1904 | 451,4621 | 0 | 0 |
| 16206 | Lrig1 | Mus musculus leucine-rich repeats and immunoglobulin-like domains 1 (Lrig1), mRNA. | 1,682102 | 0,01959167 | 2816,821 | 1674,584 | 0 | 0 |
| 116972 | 2310047D13Rik | Mus musculus RIKEN cDNA 2310047D13 gene (2310047D13Rik), mRNA. | 1,661169 | 0,0484444 | 179,7717 | 108,22 | 0 | 0 |
| 20014 | Rpn2 | Mus musculus ribophorin II (Rpn2), mRNA. | 1,653383 | 0,02465067 | 8740,162 | 5286,229 | 0 | 0 |
| 15530 | Hspg2 | PREDICTED: Mus musculus perlecan (heparan sulfate proteoglycan 2) (Hspg2), mRNA. | 1,6367 | 0,03110137 | 4036,538 | 2466,266 | 0 | 0 |
| 260409 | Cdc42ep3 | Mus musculus CDC42 effector protein (Rho GTPase binding) 3 (Cdc42ep3), mRNA. | 1,619484 | 0,04259021 | 1705,689 | 1053,23 | 0 | 0 |
| 23882 | Gadd45g | Mus musculus growth arrest and DNA-damage-inducible 45 gamma (Gadd45g), mRNA. | 1,583698 | 0,03009802 | 953,2853 | 601,9364 | 0 | 0 |
| 11670 | Aldh3a1 | Mus musculus aldehyde dehydrogenase family 3, subfamily A1 (Aldh3a1), mRNA. | 0,0418301 | 1,1898E-14 | 16,68796 | 398,9458 | 0,01253133 | 0 |
| 242894 | Actr3b | Mus musculus ARP3 actin-related protein 3 homolog B (yeast) (Actr3b), mRNA. | 0,0965865 | 5,1977E-14 | 77,97374 | 807,2941 | 0 | 0 |
| 54427 | Dnmt3l | Mus musculus DNA (cytosine-5-)-methyltransferase 3-like (Dnmt3l), transcript variant 2, mRNA. | 0,1040261 | 1,946E-11 | 28,49325 | 273,9047 | 1,25627E-05 | 0 |
| 19288 | Ptx3 | Mus musculus pentraxin related gene, mRNA | 0,1239921 | 6,4299E-06 | 69,32537 | 559,1112 | 0 | 0 |
| 16826 | Ldb2 | Mus musculus, LIM domain binding 2, mRNA | 0,1264056 | 4,5908E-05 | 11,55715 | 91,42909 | 0,02111796 | 1,25627E-05 |
| 21401 | Tcea3 | Mus musculus transcription elongation factor A (SII), 3 (Tcea3), mRNA. | 0,1287656 | 3,8499E-06 | 12,84083 | 99,7225 | 0,03759398 | 0 |
| 654812 | Angptl7 | Mus musculus angiopoietin-like 7 (Angptl7), mRNA. | 0,1446798 | 5,7359E-12 | 82,68754 | 571,521 | 0 | 0 |
| 15904 | Id4 | Mus musculus inhibitor of DNA binding 4 (Id4), mRNA. | 0,1480928 | 0,00165588 | 15,35318 | 103,6727 | 0,03007519 | 0 |
| 14620 | Gjb3 | Mus musculus gap junction protein, beta 3 (Gjb3), mRNA. | 0,1599219 | 0,01665314 | 14,852 | 92,87034 | 0,02255639 | 0 |
| 54722 | Dfna5h | Mus musculus deafness, autosomal dominant 5 homolog (human) (Dfna5h), mRNA. | 0,1682625 | 6,4169E-09 | 36,70437 | 218,1376 | 0 | 0 |
| 17873 | Gadd45b | Mus musculus growth arrest and DNA-damage-inducible 45 beta (Gadd45b), mRNA. | 0,1686197 | 1,409E-06 | 20,81783 | 123,4603 | 0,007518797 | 0 |
| 15368 | Hmox1 | Mus musculus heme oxygenase (decycling) 1 (Hmox1), mRNA. | 0,1854198 | 0,00158469 | 16,89248 | 91,104 | 0,01253133 | 0 |
| 18669 | Abcb1b | Mus musculus ATP-binding cassette, sub-family B (MDR/TAP), member 1B (Abcb1b), mRNA. | 0,186768 | 2,2011E-08 | 43,90775 | 235,0925 | 0 | 0 |
| 171508 | Creld1 | Mus musculus cysteine-rich with EGF-like domains 1 (Creld1), mRNA. | 0,1868819 | 0,00140145 | 12,65949 | 67,74061 | 0,04260652 | 0 |
| 230587 | Glis1 | Mus musculus GLIS family zinc finger 1 (Glis1), mRNA. | 0,189046 | 1,417E-06 | 53,15893 | 281,1958 | 0 | 0 |
| 18383 | Tnfrsf11b | Mus musculus tumor necrosis factor receptor superfamily, member 11b (osteoprotegerin) (Tnfrsf11b), mRNA. | 0,1902034 | 0,00635713 | 45,43797 | 238,8915 | 0 | 0 |
| 14165 | Fgf10 | Mus musculus fibroblast growth factor 10 (Fgf10), mRNA. | 0,1963378 | 7,0565E-06 | 76,09884 | 387,5914 | 0,005502499 | 3,03737E-06 |
| 71436 | Flrt3 | Mus musculus fibronectin leucine rich transmembrane protein 3 (Flrt3), mRNA. | 0,1987842 | 0,00572796 | 47,27707 | 237,8312 | 0 | 0 |
| 71839 | Osgin1 | Mus musculus oxidative stress induced growth inhibitor 1 (Osgin1), mRNA. | 0,2000516 | 5,6261E-07 | 31,36821 | 156,8006 | 0,002506266 | 0 |
| 11535 | Adm | Mus musculus adrenomedullin (Adm), mRNA. | 0,2176316 | 0,00046142 | 18,06111 | 82,98936 | 0,0112782 | 0 |
| 16156 | Il11 | Mus musculus interleukin 11 (Il11), mRNA. | 0,2437876 | 0,00416478 | 17,10521 | 70,16438 | 0,01253133 | 0 |
| 13844 | Ephb2 | Mus musculus ephrin type-B receptor 2, mRNA | 0,2489738 | 1,2336E-05 | 70,63631 | 283,7098 | 0 | 0 |
| 20512 | Slc1a3 | Mus musculus solute carrier family 1 (glial high affinity glutamate transporter), member 3 (Slc1a3), mRNA. | 0,2532443 | 3,2984E-09 | 304,3129 | 1201,658 | 0 | 0 |
| 19091 | Prkg1 | Mus musculus protein kinase, cGMP-dependent, type I (Prkg1), transcript variant 2, mRNA. | 0,2697394 | 0,03854831 | 28,19618 | 104,5312 | 0,002506266 | 0 |
| 242083 | Ppm1l | Mus musculus protein phosphatase 1 (formerly 2C)-like (Ppm1l), mRNA. | 0,2734746 | 0,00015115 | 92,76369 | 339,2041 | 0 | 0 |
| 22418 | Wnt5a | Mus musculus wingless-related MMTV integration site 5A (Wnt5a), mRNA. | 0,2748643 | 2,0969E-06 | 136,3739 | 496,1497 | 7,85171E-07 | 0 |
| 76880 | 6430411K18Rik | Mus musculus RIKEN cDNA 6430411K18 gene (6430411K18Rik), non-coding RNA. | 0,2786647 | 2,5881E-06 | 60,00273 | 215,3223 | 0 | 0 |
| 56332 | Amotl2 | Mus musculus angiomotin-like 2 (Amotl2), mRNA. | 0,2795979 | 0,00472357 | 31,1567 | 111,4339 | 0,002506266 | 0 |
| 18301 | Fxyd5 | Mus musculus FXYD domain-containing ion transport regulator 5 (Fxyd5), mRNA. | 0,2932016 | 0,03496198 | 18,51925 | 63,16218 | 0,004882535 | 1,61134E-06 |
| 69065 | Chac1 | Mus musculus ChaC, cation transport regulator-like 1 (E. coli) (Chac1), mRNA. | 0,2950769 | 0,03423304 | 54,49183 | 184,6699 | 0 | 0 |
| 13636 | Efna1 | Mus musculus ephrin A1 (Efna1), mRNA. | 0,2977356 | 0,03704397 | 27,28665 | 91,64725 | 0,002506266 | 0 |
| 13641 | Efnb1 | Mus musculus ephrin B1 (Efnb1), mRNA. | 0,2985573 | 0,01161396 | 26,66685 | 89,31902 | 0,002506266 | 0 |
| 23792 | Adam23 | Mus musculus a disintegrin and metallopeptidase domain 23 (Adam23), mRNA. | 0,3021842 | 0,02542158 | 880,9091 | 2915,139 | 0 | 0 |
| 227929 | Cytip | Mus musculus cytohesin 1 interacting protein (Cytip), mRNA. | 0,303528 | 4,866E-06 | 121,5612 | 400,4941 | 0 | 0 |
| 213649 | Arhgef19 | Mus musculus Rho guanine nucleotide exchange factor (GEF) 19 (Arhgef19), mRNA. | 0,3053322 | 0,01219886 | 25,97109 | 85,05849 | 0,002506266 | 0 |
| 13849 | Ephx1 | Mus musculus epoxide hydrolase 1, microsomal (Ephx1), mRNA. | 0,3080072 | 6,251E-07 | 432,0109 | 1402,6 | 0 | 0 |
| 78785 | Clip4 | Mus musculus CAP-GLY domain containing linker protein family, member 4 (Clip4), mRNA. | 0,3129856 | 5,6965E-05 | 51,36002 | 164,0971 | 0 | 0 |
| 12575 | Cdkn1a | Mus musculus cyclin-dependent kinase inhibitor 1A (P21) (Cdkn1a), mRNA. | 0,3141907 | 2,2158E-05 | 149,7902 | 476,7493 | 3,27975E-10 | 0 |
| 56533 | Rgs17 | Mus musculus regulator of G-protein signaling 17, mRNA | 0,3172472 | 4,3687E-05 | 56,14568 | 176,9777 | 0 | 0 |
| 66403 | Asf1a | Mus musculus ASF1 anti-silencing function 1 homolog A (S. cerevisiae) (Asf1a), mRNA. | 0,3184932 | 0,0355262 | 25,5697 | 80,28335 | 0,003759399 | 0 |
| 14998 | H2-DMa | Mus musculus histocompatibility 2, class II, locus DMa (H2-DMa), mRNA. | 0,3215483 | 0,0007395 | 50,03795 | 155,6156 | 0 | 0 |
| 14456 | Gas6 | Mus musculus growth arrest specific 6 (Gas6), mRNA. | 0,3225032 | 1,111E-05 | 142,9073 | 443,1191 | 0 | 0 |
| 107227 | Macrod1 | Mus musculus MACRO domain containing 1 (Macrod1), mRNA. | 0,3228004 | 0,00022131 | 45,09617 | 139,703 | 0 | 0 |
| 109672 | Cyb5 | Mus musculus cytochrome b-5 (Cyb5), mRNA. | 0,3237753 | 3,4128E-05 | 205,2708 | 633,9914 | 0 | 0 |
| 66895 | 1300014I06Rik | Mus musculus RIKEN cDNA 1300014I06 gene (1300014I06Rik), mRNA. | 0,3278553 | 0,03787176 | 20,53413 | 62,63168 | 0,00877193 | 0 |
| 13390 | Dlx1 | Mus musculus distal-less homeobox 1 (Dlx1), mRNA. | 0,3279544 | 0,00499729 | 156,4497 | 477,0472 | 0 | 0 |
| 14205 | Figf | Mus musculus c-fos induced growth factor (Figf), mRNA. | 0,33403 | 9,5711E-06 | 309,16 | 925,5456 | 0 | 0 |
| 15200 | Hbegf | Mus musculus heparin-binding EGF-like growth factor (Hbegf), mRNA. | 0,3360107 | 0,00499558 | 36,71506 | 109,2675 | 0,001253133 | 0 |
| 14630 | Gclm | Mus musculus glutamate-cysteine ligase , modifier subunit (Gclm), mRNA. | 0,3407409 | 0,00013421 | 269,2775 | 790,2705 | 0 | 0 |
| 67760 | Slc38a2 | Mus musculus solute carrier family 38, member 2 (Slc38a2), mRNA. | 0,3430178 | 0,00131878 | 564,2791 | 1645,043 | 0,000498808 | 0,001611339 |
| 74137 | Nuak2 | Mus musculus NUAK family, SNF1-like kinase, 2 (Nuak2), mRNA. | 0,3454098 | 5,4066E-06 | 144,2404 | 417,592 | 0 | 0 |
| 105298 | Epdr1 | Mus musculus ependymin related protein 1 (zebrafish) (Epdr1), mRNA. | 0,3510638 | 8,674E-06 | 135,1488 | 384,9694 | 0 | 0 |
| 24088 | Tlr2 | Mus musculus toll-like receptor 2 (Tlr2), mRNA. | 0,3520601 | 1,7026E-05 | 150,7605 | 428,2238 | 0 | 0 |
| 107449 | Unc5b | Mus musculus unc-5 homolog B (C. elegans) (Unc5b), mRNA. | 0,3548568 | 0,00869638 | 37,15366 | 104,7004 | 0 | 0 |
| 14461 | Gata2 | Mus musculus GATA binding protein 2 (Gata2), mRNA. | 0,3565561 | 4,0663E-06 | 225,6547 | 632,873 | 0 | 0 |
| 267019 | Rps15a | Mus musculus ribosomal protein S15a (Rps15a), mRNA. | 0,3587208 | 0,00007683 | 80,31608 | 223,8958 | 0 | 0 |
| 74007 | Btbd11 | Mus musculus BTB (POZ) domain containing 11 (Btbd11), transcript variant 2, mRNA. | 0,363129 | 0,00017956 | 69,82734 | 192,2935 | 0 | 0 |
| 67739 | 4930570C03Rik | Mus musculus RIKEN cDNA 4930570C03 gene (4930570C03Rik), mRNA. | 0,3704954 | 3,9047E-06 | 577,7795 | 1559,478 | 0 | 0 |
| 59010 | Sqrdl | Mus musculus sulfide quinone reductase-like (yeast) (Sqrdl), mRNA. | 0,3719745 | 0,00150469 | 50,37995 | 135,4393 | 0 | 0 |
| 433752 | AA415398 | Mus musculus expressed sequence AA415398 (AA415398), mRNA. | 0,3725354 | 0,00116282 | 54,56543 | 146,4704 | 0 | 0 |
| 68203 | Diras2 | Mus musculus DIRAS family, GTP-binding RAS-like 2 (Diras2), mRNA. | 0,3733634 | 3,0937E-05 | 133,1684 | 356,6724 | 0 | 0 |
| 16904 | Gzmm | Mus musculus granzyme M (lymphocyte met-ase 1) (Gzmm), mRNA. | 0,3759067 | 0,04133955 | 26,9388 | 71,66352 | 0,002506266 | 0 |
| 20716 | Serpina3n | Mus musculus serine (or cysteine) peptidase inhibitor, clade A, member 3N (Serpina3n), mRNA. | 0,376683 | 6,8881E-06 | 357,0518 | 947,8839 | 0 | 0 |
| 54635 | Pdgfc | Mus musculus platelet-derived growth factor, C polypeptide (Pdgfc), mRNA. | 0,3770622 | 0,00150469 | 84,34894 | 223,7003 | 0 | 0 |
| 21366 | Slc6a6 | Mus musculus solute carrier family 6 (neurotransmitter transporter, taurine), member 6 (Slc6a6), mRNA. | 0,3782048 | 3,3793E-06 | 2157,877 | 5705,578 | 0 | 0 |
| 55984 | Camkk1 | Mus musculus calcium/calmodulin-dependent protein kinase kinase 1, alpha (Camkk1), mRNA. | 0,3868585 | 0,00498693 | 44,54494 | 115,1453 | 0 | 0 |
| 55963 | Slc1a4 | Mus musculus solute carrier family 1 (glutamate/neutral amino acid transporter), member 4 (Slc1a4), mRNA. | 0,3891371 | 2,8423E-05 | 203,7001 | 523,4661 | 0 | 0 |
| 76156 | 6330503C03Rik | Mus musculus RIKEN cDNA 6330503C03 gene (6330503C03Rik), mRNA. | 0,3924941 | 0,00044959 | 120,1619 | 306,1494 | 0 | 0 |
| 320080 | Zbtb39 | Mus musculus zinc finger and BTB domain containing 39 (Zbtb39), mRNA. | 0,4008877 | 0,01003963 | 70,04984 | 174,7368 | 0 | 0 |
| 18574 | Pde1b | Mus musculus phosphodiesterase 1B, Ca2+-calmodulin dependent (Pde1b), mRNA. | 0,4023464 | 0,01476451 | 39,77571 | 98,85938 | 0 | 0 |
| 72400 | 2610028A01Rik | Mus musculus RIKEN cDNA 2610028A01 gene (2610028A01Rik), mRNA. | 0,4048581 | 0,01165598 | 82,18085 | 202,9868 | 0 | 0 |
| 13723 | Emb | Mus musculus embigin (Emb), mRNA. | 0,4090246 | 1,8546E-05 | 1490,012 | 3642,841 | 0 | 0 |
| 14866 | Gstm5 | Mus musculus glutathione S-transferase, mu 5 (Gstm5), mRNA. | 0,4175841 | 0,00075069 | 636,7589 | 1524,864 | 0 | 0 |
| 16498 | Kcnab2 | Mus musculus potassium voltage-gated channel, shaker-related subfamily, beta member 2 (Kcnab2), mRNA. | 0,417909 | 0,00427208 | 78,91422 | 188,8311 | 0 | 0 |
| 24059 | Slco2a1 | Mus musculus solute carrier organic anion transporter family, member 2a1 (Slco2a1), mRNA. | 0,4184846 | 0,0001829 | 164,5432 | 393,1882 | 0 | 0 |
| 216363 | Rab3ip | Mus musculus RAB3A interacting protein (Rab3ip), mRNA. | 0,4185389 | 0,01835551 | 42,74945 | 102,1397 | 0 | 0 |
| 19736 | Rgs4 | Mus musculus regulator of G-protein signaling 4 (Rgs4), mRNA. | 0,4190681 | 0,02075136 | 363,8257 | 868,178 | 0 | 0 |
| 13610 | Edg3 | Mus musculus endothelial differentiation, sphingolipid G-protein-coupled receptor, 3 (Edg3), mRNA. | 0,4200806 | 0,00370767 | 63,22589 | 150,509 | 0 | 0 |
| 18645 | Pfn2 | Mus musculus profilin 2 (Pfn2), mRNA. | 0,420812 | 0,00399573 | 537,9476 | 1278,356 | 7,99897E-06 | 4,36534E-07 |
| 20324 | Sdpr | Mus musculus serum deprivation response (Sdpr), mRNA. | 0,4218273 | 0,00010056 | 1199,695 | 2844,044 | 0 | 0 |
| 105037 | AW555464 | Mus musculus expressed sequence AW555464, mRNA | 0,4239979 | 0,00093908 | 297,3801 | 701,3717 | 0 | 0 |
| 677317 | LOC677317 | PREDICTED: Mus musculus similar to Mod1 protein, transcript variant 4 (LOC677317), mRNA. | 0,4240453 | 0,03001361 | 87,56762 | 206,5053 | 0 | 0 |
| 14664 | Slc6a9 | Mus musculus solute carrier family 6 (neurotransmitter transporter, glycine), member 9 (Slc6a9), mRNA. | 0,4247285 | 8,1348E-05 | 358,8861 | 844,9776 | 0 | 0 |
| 67017 | 2010011I20Rik | Mus musculus RIKEN cDNA 2010011I20 gene (2010011I20Rik), mRNA. | 0,4263265 | 0,00356683 | 74,03358 | 173,6546 | 0 | 0 |
| 21844 | Tiam1 | Mus musculus T-cell lymphoma invasion and metastasis 1 (Tiam1), mRNA. | 0,4329965 | 0,0002137 | 224,4014 | 518,2522 | 0 | 0 |
| 21859 | Timp3 | Mus musculus tissue inhibitor of metalloproteinase 3 (Timp3), mRNA. | 0,434107 | 0,00410843 | 1012,864 | 2333,212 | 0 | 0 |
| 16173 | Il18 | Mus musculus interleukin 18 (Il18), mRNA. | 0,4343607 | 0,01017944 | 112,266 | 258,4626 | 0 | 0 |
| 20620 | Plk2 | Mus musculus polo-like kinase 2 (Drosophila) (Plk2), mRNA. | 0,4352018 | 0,00672799 | 97,65088 | 224,3807 | 0 | 0 |
| 56738 | Mocs1 | Mus musculus molybdenum cofactor synthesis 1 (Mocs1), transcript variant 1, mRNA. | 0,4357502 | 8,7083E-05 | 763,6218 | 1752,43 | 0 | 0 |
| 20509 | Slc19a1 | Mus musculus solute carrier family 19 (sodium/hydrogen exchanger), member 1 (Slc19a1), mRNA. | 0,4426794 | 0,00100926 | 149,5435 | 337,8145 | 0 | 0 |
| 22337 | Vdr | Mus musculus vitamin D receptor (Vdr), mRNA. | 0,4431811 | 0,02625027 | 30,46648 | 68,74499 | 4,36534E-07 | 0,005966331 |
| 58859 | Efemp2 | Mus musculus epidermal growth factor-containing fibulin-like extracellular matrix protein 2 (Efemp2), mRNA. | 0,4439086 | 0,00010406 | 1368,213 | 3082,196 | 0 | 0 |
| 109901 | Ela1 | Mus musculus elastase 1, pancreatic (Ela1), mRNA. | 0,4447165 | 0,0020524 | 119,024 | 267,6402 | 0 | 0 |
| 12479 | Cd1d1 | Mus musculus CD1d1 antigen (Cd1d1), mRNA. | 0,4454058 | 0,00070973 | 174,833 | 392,5251 | 0 | 0 |
| 668171 | Zxda | Mus musculus zinc finger, X-linked, duplicated A (Zxda), non-coding RNA. | 0,4461677 | 0,01331485 | 99,31958 | 222,6059 | 0 | 0 |
| 116914 | Slc19a2 | Mus musculus solute carrier family 19 (thiamine transporter), member 2 (Slc19a2), mRNA. | 0,4487716 | 0,00124616 | 310,3611 | 691,5792 | 0 | 0 |
| 11785 | Apbb1 | Mus musculus amyloid beta (A4) precursor protein-binding, family B, member 1 (Apbb1), mRNA. | 0,4487991 | 0,00324718 | 90,25184 | 201,0963 | 0 | 0 |
| 171095 | Il17rc | Mus musculus interleukin 17 receptor C (Il17rc), mRNA. | 0,4495671 | 0,00458527 | 240,7224 | 535,4536 | 0 | 0 |
| 108089 | Rnf144a | Mus musculus ring finger protein 144A (Rnf144a), transcript variant 2, mRNA. | 0,4502371 | 0,00633046 | 216,5547 | 480,9792 | 0 | 0 |
| 14860 | Gsta4 | Mus musculus glutathione S-transferase, alpha 4 (Gsta4), mRNA. | 0,4565791 | 0,00108647 | 543,0723 | 1189,438 | 0 | 0 |
| 68591 | Mocos | Mus musculus molybdenum cofactor sulfurase (Mocos), mRNA. | 0,4586131 | 0,00318816 | 110,7125 | 241,4073 | 0 | 0 |
| 55989 | Nol5 | Mus musculus nucleolar protein 5 (Nol5), mRNA. | 0,4613019 | 0,02853181 | 1068,054 | 2315,304 | 0 | 0 |
| 19009 | Pou6f1 | Mus musculus POU domain, class 6, transcription factor 1 (Pou6f1), mRNA. | 0,4638636 | 0,00144752 | 162,2994 | 349,8861 | 0 | 0 |
| 16948 | Lox | Mus musculus lysyl oxidase (Lox), mRNA. | 0,4645865 | 0,00024956 | 2709,675 | 5832,444 | 0 | 0 |
| 170787 | Hdac10 | Mus musculus histone deacetylase 10 (Hdac10), mRNA. | 0,4682001 | 0,03163427 | 54,54498 | 116,4993 | 0 | 0 |
| 232232 | Hdac11 | Mus musculus histone deacetylase 11 (Hdac11), mRNA. | 0,469431 | 0,04331632 | 57,02908 | 121,4855 | 0 | 0 |
| 23872 | Ets2 | Mus musculus E26 avian leukemia oncogene 2, 3' domain (Ets2), mRNA. | 0,4713942 | 0,01160365 | 748,1522 | 1587,105 | 0 | 0 |
| 13386 | Dlk1 | Mus musculus delta-like 1 homolog (Drosophila) (Dlk1), mRNA. | 0,4751098 | 0,00093908 | 10043,98 | 21140,34 | 0 | 0 |
| 225870 | Rin1 | Mus musculus Ras and Rab interactor 1 (Rin1), mRNA. | 0,4752529 | 0,00111504 | 267,3327 | 562,5062 | 0 | 0 |
| 20471 | Six1 | Mus musculus sine oculis-related homeobox 1 homolog (Drosophila) (Six1), mRNA. | 0,4797631 | 0,00489256 | 120,6656 | 251,5109 | 0 | 0 |
| 59053 | Brp16 | Mus musculus brain protein 16 (Brp16), mRNA. | 0,4822294 | 0,03325379 | 167,514 | 347,3741 | 0,000706654 | 0,000153894 |
| 11669 | Aldh2 | Mus musculus aldehyde dehydrogenase 2, mitochondrial (Aldh2), nuclear gene encoding mitochondrial protein, mRNA. | 0,4868123 | 0,00170348 | 1696,808 | 3485,55 | 0 | 0 |
| 80888 | Hspb8 | Mus musculus heat shock protein 8 (Hspb8), mRNA. | 0,488326 | 0,00145762 | 352,0595 | 720,9517 | 0 | 0 |
| 15404 | Hoxa7 | Mus musculus homeo box A7 (Hoxa7), mRNA. | 0,4921415 | 0,01848367 | 843,9711 | 1714,895 | 0 | 0 |
| 107765 | Ankrd1 | Mus musculus ankyrin repeat domain 1 (cardiac muscle) (Ankrd1), mRNA. | 0,4962667 | 0,0169719 | 123,3187 | 248,4927 | 0 | 0 |
| 76927 | 1700021C14Rik | Mus musculus RIKEN cDNA 1700021C14 gene (1700021C14Rik), mRNA. | 0,4973733 | 0,00489256 | 169,151 | 340,0886 | 0 | 0 |
| 70358 | Steap1 | Mus musculus six transmembrane epithelial antigen of the prostate 1 (Steap1), mRNA. | 0,5006 | 0,02612559 | 79,21106 | 158,2322 | 0 | 0 |
| 64817 | Svep1 | Mus musculus sushi, von Willebrand factor type A, EGF and pentraxin domain containing 1 (Svep1), mRNA. | 0,5023032 | 0,02026491 | 100,123 | 199,3278 | 0 | 0 |
| 22401 | Zmat3 | Mus musculus zinc finger matrin type 3 (Zmat3), mRNA. | 0,5035527 | 0,00194815 | 642,5668 | 1276,067 | 0 | 0 |
| 11854 | Rhod | Mus musculus ras homolog gene family, member D (Rhod), mRNA. | 0,5048224 | 0,04254982 | 341,2558 | 675,9917 | 0,003738199 | 0,002205545 |
| 66532 | 2210417D09Rik | Mus musculus RIKEN cDNA 2210417D09 gene (2210417D09Rik), mRNA. | 0,5051467 | 0,02560896 | 143,2896 | 283,6593 | 0 | 0 |
| 22042 | Tfrc | Mus musculus transferrin receptor (Tfrc), mRNA. | 0,505383 | 0,04247516 | 176,9169 | 350,065 | 0 | 0 |
| 12237 | Bub3 | Mus musculus budding uninhibited by benzimidazoles 3 homolog (S. cerevisiae) (Bub3), mRNA. | 0,5074649 | 0,02952083 | 142,9005 | 281,5969 | 0 | 0 |
| 243912 | Hspb6 | Mus musculus heat shock protein, alpha-crystallin-related, B6 (Hspb6), mRNA. | 0,5105127 | 0,00366543 | 336,1583 | 658,472 | 0 | 0 |
| 246154 | Vasn | Mus musculus vasorin (Vasn), mRNA. | 0,511288 | 0,00184665 | 2069,338 | 4047,303 | 0 | 0 |
| 216188 | Aldh1l2 | Mus musculus aldehyde dehydrogenase 1 family, member L2 (Aldh1l2), mRNA. | 0,5127124 | 0,02056986 | 208,0685 | 405,8191 | 0 | 0 |
| 226421 | 5430435G22Rik | Mus musculus RIKEN cDNA 5430435G22 gene (5430435G22Rik), mRNA. | 0,518461 | 0,0112699 | 370,0544 | 713,7556 | 0 | 0 |
| 72042 | Cotl1 | Mus musculus coactosin-like 1 (Dictyostelium) (Cotl1), mRNA. | 0,5195887 | 0,01975094 | 480,6915 | 925,1385 | 0 | 0 |
| 19679 | Pitpnm2 | Mus musculus phosphatidylinositol transfer protein, membrane-associated 2 (Pitpnm2), mRNA. | 0,5232068 | 0,01691748 | 140,8815 | 269,2654 | 0 | 0 |
| 12696 | Cirbp | Mus musculus cold inducible RNA binding protein (Cirbp), mRNA. | 0,5233102 | 0,00966686 | 273,6898 | 522,9972 | 0 | 0 |
| 68404 | Nrn1 | Mus musculus neuritin 1 (Nrn1), mRNA. | 0,5238764 | 0,00916374 | 979,4315 | 1869,585 | 0 | 0 |
| 211666 | Mgst2 | Mus musculus microsomal glutathione S-transferase 2 (Mgst2), mRNA. | 0,5253773 | 0,01779643 | 489,1796 | 931,1016 | 0 | 0 |
| 170460 | Stard5 | Mus musculus StAR-related lipid transfer (START) domain containing 5 (Stard5), mRNA. | 0,5255257 | 0,01832625 | 215,0281 | 409,1677 | 0 | 0 |
| 19934 | Rpl22 | Mus musculus ribosomal protein L22 (Rpl22), mRNA. | 0,5258135 | 0,00324718 | 2111,962 | 4016,561 | 0 | 0 |
| 68067 | 3010026O09Rik | Mus musculus RIKEN cDNA 3010026O09 gene (3010026O09Rik), mRNA. | 0,5297533 | 0,02075136 | 131,3788 | 247,9999 | 0 | 0 |
| 13803 | Enc1 | Mus musculus ectodermal-neural cortex 1 (Enc1), mRNA. | 0,5364504 | 0,00671902 | 535,3694 | 997,985 | 0 | 0 |
| 67041 | Oxct1 | Mus musculus 3-oxoacid CoA transferase 1 (Oxct1), mRNA. | 0,5370609 | 0,00669488 | 1982,746 | 3691,845 | 0 | 0 |
| 13078 | Cyp1b1 | Mus musculus cytochrome P450, family 1, subfamily b, polypeptide 1 (Cyp1b1), mRNA. | 0,5383847 | 0,01837555 | 171,5853 | 318,7039 | 0 | 0 |
| 19294 | Pvrl2 | Mus musculus poliovirus receptor-related 2 (Pvrl2), mRNA. | 0,5387915 | 0,03937874 | 159,6534 | 296,3176 | 7,2056E-07 | 8,2009E-05 |
| 230673 | Ipo13 | Mus musculus importin 13 (Ipo13), mRNA. | 0,5396584 | 0,00550598 | 1764,157 | 3269,026 | 0 | 0 |
| 107587 | Osr2 | Mus musculus odd-skipped related 2 (Drosophila) (Osr2), mRNA. | 0,5404629 | 0,00681448 | 710,4856 | 1314,587 | 0 | 0 |
| 19359 | Rad23b | Mus musculus RAD23b homolog (S. cerevisiae) (Rad23b), mRNA. | 0,54058 | 0,0241455 | 330,4821 | 611,3472 | 0 | 0 |
| 230793 | Ahdc1 | Mus musculus AT hook, DNA binding motif, containing 1 (Ahdc1), mRNA. | 0,5407891 | 0,03828454 | 183,4628 | 339,2502 | 0 | 0 |
| 70155 | Ogfrl1 | Mus musculus opioid growth factor receptor-like 1 (Ogfrl1), mRNA. | 0,5430804 | 0,01523567 | 222,9346 | 410,5001 | 0 | 0 |
| 105037 | 2610020C11Rik | Mus musculus RIKEN cDNA 2610020C11Rik gene (2610020C11Rik), mRNA | 0,5431743 | 0,02383407 | 258,2432 | 475,4335 | 0 | 0 |
| 110208 | Pgd | Mus musculus phosphogluconate dehydrogenase, mRNA | 0,5440258 | 0,01589124 | 1374,267 | 2526,106 | 0 | 0 |
| 233016 | Blvrb | Mus musculus biliverdin reductase B (flavin reductase (NADPH)) (Blvrb), mRNA. | 0,5462692 | 0,00671902 | 2185,997 | 4001,684 | 0 | 0 |
| 223775 | Pim3 | Mus musculus proviral integration site 3, mRNA | 0,5488914 | 0,02214577 | 186,6161 | 339,9872 | 0 | 0 |
| 15402 | Hoxa5 | Mus musculus homeobox A5, mRNA | 0,5521509 | 0,00852807 | 1982,794 | 3591,037 | 0 | 0 |
| 12608 | Cebpb | Mus musculus CCAAT/enhancer binding protein (C/EBP), beta (Cebpb), mRNA. | 0,5647852 | 0,02040645 | 2164,845 | 3833,042 | 0 | 0 |
| 100494 | Zfand2a | Mus musculus zinc finger, AN1-type domain 2A (Zfand2a), mRNA. | 0,5743169 | 0,02239435 | 516,2742 | 898,9361 | 0 | 0 |
| 105827 | Amigo2 | Mus musculus adhesion molecule with Ig like domain 2 (Amigo2), mRNA. | 0,576241 | 0,03401132 | 267,213 | 463,7176 | 0 | 0 |
| 28088 | D10Wsu52e | Mus musculus DNA segment, Chr 10, Wayne State University 52, expressed (D10Wsu52e), mRNA. | 0,576374 | 0,02114385 | 728,0728 | 1263,195 | 0 | 0 |
| 22022 | Tpst2 | Mus musculus protein-tyrosine sulfotransferase 2 (Tpst2), mRNA. | 0,5808827 | 0,0252558 | 630,1226 | 1084,767 | 0 | 0 |
| 207785 | Csrnp2 | Mus musculus cysteine-serine-rich nuclear protein 2 (Csrnp2), mRNA. | 0,5818284 | 0,03052973 | 415,8866 | 714,7925 | 0 | 0 |
| 19181 | Psmc2 | Mus musculus proteasome (prosome, macropain) 26S subunit, ATPase 2 (Psmc2), mRNA. | 0,59675 | 0,03052973 | 4236,777 | 7099,752 | 0 | 0 |
| 11931 | Atp1b1 | Mus musculus ATPase, Na+/K+ transporting, beta 1 polypeptide (Atp1b1), mRNA. | 0,6049048 | 0,04779637 | 696,6985 | 1151,749 | 0 | 0 |
| 240514 | Ccdc85b | Mus musculus coiled-coil domain containing 85B (Ccdc85b), mRNA. | 0,6155602 | 0,00056718 | 358,9534 | 583,1328 | 3,14068E-06 | 0 |
