# Supplementary material for: Growth Factor PDGF-BB Stimulates Cultured Cardiomyocytes to Synthesize the Extracellular Matrix Component Hyaluronan
Source: PLoS One. 2010 Dec 21;5(12):e14393. doi: 10.1371/journal.pone.0014393 (PMC3006157; doi:10.1371/journal.pone.0014393)
Supplement: Table S2 — Differentially expressed genes in fibroblasts cultured in media supernatant from ultrcentrifugation, previously incubated with cardiomyocytes. (0.16 MB DOC) [file pone.0014393.s002.doc]

| Table S2. Differentially expressed genes in fibroblasts cultured in media supernatant from ultrcentrifugation, previously incubated with cardiomyocytes. | | | | | | | | |
| --- | --- | --- | --- | --- | --- | --- | --- | --- |
| ENTREZ GENE ID | SYMBOL | DEFINITION | Foldchange | Diff *P*-value | Treated cells average signal | Control average signal | Treated cells detection *P*-value | Control detection *P*-value |
| 72713 | Angptl1 | Mus musculus angiopoietin-like 1 (Angptl1), mRNA. | 16,90789 | 0,00116933 | 63,74968 | 3,77041 | 0 | 0 |
| 56096 | Plac1 | Mus musculus placental specific protein 1 (Plac1), mRNA. | 5,464256 | 0,00100786 | 147,6323 | 27,01783 | 0 | 0 |
| 30959 | Ddx25 | Mus musculus DEAD (Asp-Glu-Ala-Asp) box polypeptide 25 (Ddx25), mRNA. | 3,930568 | 0,00064414 | 284,6519 | 72,42003 | 0 | 0 |
| 217066 | OTTMUSG00000025408 | Mus musculus predicted gene, OTTMUSG00000025408 (OTTMUSG00000025408), non-coding RNA. | 3,776911 | 0,00349216 | 356,913 | 94,49866 | 0 | 0 |
| 240913 | Adamts4 | Mus musculus a disintegrin-like and metallopeptidase (reprolysin type) with thrombospondin type 1 motif, 4, mRNA | 3,751177 | 0,00144452 | 161,596 | 43,07874 | 0 | 0 |
| 100046120 | LOC100046120 | PREDICTED: Mus musculus similar to clusterin (LOC100046120), mRNA. | 3,648818 | 0,00074883 | 1502,397 | 411,7488 | 0 | 0 |
| 100048554 | LOC100048554 | PREDICTED: Mus musculus similar to monocyte chemoattractant protein-2 (MCP-2)  (LOC100048554), mRNA. | 3,62305 | 0,00100786 | 363,3379 | 100,2851 | 0 | 0 |
| 217066 | BC099439 | Mus musculus cDNA sequence BC099439 (BC099439), mRNA. | 3,250162 | 0,00384202 | 349,4912 | 107,5304 | 0 | 0 |
| 232174 | Cyp26b1 | Mus musculus cytochrome P450, family 26, subfamily b, polypeptide 1 (Cyp26b1), mRNA. | 3,249254 | 0,00252976 | 178,8673 | 55,04873 | 0 | 0 |
| 192156 | Mvd | Mus musculus mevalonate (diphospho) decarboxylase (Mvd), mRNA. | 3,248109 | 0,00017888 | 655,93 | 201,9421 | 0 | 0 |
| 68728 | Trp53inp2 | Mus musculus transformation related protein 53 inducible nuclear protein 2 (Trp53inp2), mRNA. | 2,922155 | 0,04068745 | 120,8532 | 41,35757 | 0 | 0 |
| 21808 | Tgfb2 | Mus musculus transforming growth factor, beta 2 (Tgfb2), mRNA. | 2,800022 | 0,0011142 | 423,2532 | 151,1607 | 0 | 0 |
| 72324 | Plxdc1 | Mus musculus plexin domain containing 1 (Plxdc1), mRNA. | 2,78897 | 0,01702422 | 155,8219 | 55,87078 | 0 | 0 |
| 110196 | Fdps | Mus musculus farnesyl diphosphate synthetase (Fdps), mRNA. | 2,45351 | 0,00129737 | 7417,304 | 3023,14 | 0 | 0 |
| 13197 | Gadd45a | Mus musculus growth arrest and DNA-damage-inducible 45 alpha (Gadd45a), mRNA. | 2,396186 | 0,00347964 | 1044,978 | 436,1004 | 0 | 0 |
| 207521 | Dtx4 | Mus musculus deltex 4 homolog (Drosophila) (Dtx4), mRNA. XM_001000490 | 2,387558 | 0,02631126 | 413,0169 | 172,9872 | 0 | 0 |
| 20356 | Sema5a | Mus musculus sema domain, seven thrombospondin repeats (type 1 and type 1-like), transmembrane domain (TM) and short cytoplasmic domain, (semaphorin) 5A (Sema5a), mRNA. | 2,146626 | 0,02208199 | 699,8361 | 326,0168 | 0 | 0 |
| 17965 | Nbl1 | Mus musculus neuroblastoma, suppression of tumorigenicity 1 (Nbl1), mRNA. | 2,137576 | 0,04698921 | 323,4763 | 151,3286 | 0 | 0 |
| 11541 | Adora2b | Mus musculus adenosine A2b receptor (Adora2b), mRNA. | 2,116608 | 0,01238456 | 1328,307 | 627,5641 | 0 | 0 |
| 20250 | Scd2 | Mus musculus stearoyl-Coenzyme A desaturase 2 (Scd2), mRNA. | 2,096728 | 0,02177109 | 619,0258 | 295,2342 | 0 | 0 |
| 23966 | Odz4 | Mus musculus odd Oz/ten-m homolog 4 (Drosophila) (Odz4), mRNA. | 2,052786 | 0,04068745 | 427,2348 | 208,1244 | 0 | 0 |
| 11670 | Aldh3a1 | Mus musculus aldehyde dehydrogenase family 3, subfamily A1 (Aldh3a1), mRNA. | 0,2040955 | 1,5327E-08 | 76,82644 | 376,424 | 0 | 0 |
| 68026 | 2810417H13Rik | Mus musculus RIKEN cDNA 2810417H13 gene (2810417H13Rik), mRNA. | 0,2105432 | 0,00349216 | 17,42089 | 82,74261 | 0,02506266 | 0 |
| 19092 | Prkg2 | Mus musculus protein kinase, cGMP-dependent, type II (Prkg2), mRNA. | 0,241487 | 1,9614E-06 | 248,3274 | 1028,326 | 0 | 0 |
| 66336 | Cenpp | Mus musculus centromere protein P (Cenpp), mRNA. | 0,2512386 | 0,04342204 | 30,96504 | 123,2495 | 0,002506266 | 0 |
| 71988 | Esco2 | Mus musculus establishment of cohesion 1 homolog 2 (S. cerevisiae) (Esco2), mRNA. | 0,2565683 | 0,00100786 | 33,00571 | 128,6429 | 0,001253133 | 0 |
| 218581 | Depdc1b | Mus musculus DEP domain containing 1B (Depdc1b), mRNA. | 0,2569999 | 0,00031947 | 30,23897 | 117,6614 | 0,003759399 | 0 |
| 105988 | Espl1 | Mus musculus extra spindle poles-like 1 (S. cerevisiae) (Espl1), mRNA. | 0,3123187 | 0,00336295 | 47,66616 | 152,6203 | 0 | 0 |
| 20198 | S100a4 | Mus musculus S100 calcium binding protein A4 (S100a4), mRNA. | 0,3227029 | 0,00017888 | 213,1232 | 660,4316 | 0 | 0 |
| 52033 | Pbk | Mus musculus PDZ binding kinase (Pbk), mRNA. | 0,3247704 | 1,1485E-07 | 406,5335 | 1251,757 | 0 | 0 |
| 108912 | Cdca2 | Mus musculus cell division cycle associated 2 (Cdca2), mRNA. | 0,3260603 | 1,406E-06 | 155,0774 | 475,6096 | 0 | 0 |
| 102920 | Cenpi | Mus musculus centromere protein I (Cenpi), mRNA. | 0,3463531 | 0,00067635 | 55,6611 | 160,7062 | 0 | 0 |
| 106344 | Rfc4 | Mus musculus replication factor C (activator 1) 4, 37kDa, mRNA | 0,3509216 | 0,02349151 | 35,34787 | 100,7287 | 0,0122683 | 0 |
| 97165 | Hmgb2 | Mus musculus high mobility group box 2 (Hmgb2), mRNA. | 0,3530259 | 3,5896E-05 | 112,0169 | 317,3049 | 0 | 0 |
| 60530 | Fignl1 | Mus musculus fidgetin-like 1 (Fignl1), mRNA. | 0,3591278 | 8,9209E-05 | 97,69753 | 272,0411 | 0 | 0 |
| 70385 | Ccdc99 | Mus musculus coiled-coil domain containing 99 (Ccdc99), mRNA. | 0,3605726 | 0,00209061 | 87,97317 | 243,9819 | 0 | 0 |
| 75317 | 4930547N16Rik | Mus musculus RIKEN cDNA 4930547N16 gene (4930547N16Rik), mRNA. | 0,363207 | 0,00380727 | 46,41787 | 127,8 | 0 | 0 |
| 56150 | Mad2l1 | Mus musculus MAD2 (mitotic arrest deficient, homolog)-like 1 (yeast) (Mad2l1), mRNA. | 0,3693662 | 0,00192284 | 329,6167 | 892,3845 | 0 | 0 |
| 70024 | Mcm10 | Mus musculus minichromosome maintenance deficient 10 (S. cerevisiae) (Mcm10), mRNA. | 0,3753484 | 0,00278606 | 90,58096 | 241,325 | 2,82662E-05 | 0 |
| 16571 | Kif4 | Mus musculus kinesin family member 4 (Kif4), mRNA. | 0,3767765 | 0,01677175 | 62,57717 | 166,0856 | 8,85531E-09 | 0 |
| 17427 | Mns1 | Mus musculus meiosis-specific nuclear structural protein 1 (Mns1), mRNA. | 0,3807024 | 0,00215392 | 70,24738 | 184,5204 | 0 | 0 |
| 67849 | Cdca5 | Mus musculus cell division cycle associated 5 (Cdca5), mRNA. | 0,3866171 | 0,009039 | 50,72301 | 131,197 | 0 | 0 |
| 72119 | Tpx2 | Mus musculus TPX2, microtubule-associated protein homolog (Xenopus laevis) (Tpx2), mRNA. | 0,3886143 | 0,01103901 | 47,69893 | 122,7411 | 0 | 0 |
| 14841 | Gsg2 | Mus musculus germ cell-specific gene 2 (Gsg2), mRNA. | 0,3913514 | 0,00025814 | 113,3934 | 289,7483 | 0 | 0 |
| 109212 | 6720460F02Rik | Mus musculus RIKEN cDNA 6720460F02 gene (6720460F02Rik), mRNA. | 0,3926106 | 0,02727714 | 42,18671 | 107,4518 | 0 | 0 |
| 16551 | Kif11 | Mus musculus kinesin family member 11 (Kif11), mRNA. | 0,3981343 | 0,00055185 | 116,05 | 291,4845 | 0 | 0 |
| 106795 | Tcf19 | Mus musculus transcription factor 19 (Tcf19), mRNA. | 0,4021311 | 0,00308963 | 302,433 | 752,0756 | 0 | 0 |
| 110454 | Ly6a | Mus musculus lymphocyte antigen 6 complex, locus A (Ly6a), mRNA. | 0,402619 | 8,9209E-05 | 412,1175 | 1023,592 | 0 | 0 |
| 268697 | Ccnb1 | Mus musculus cyclin B1 (Ccnb1), mRNA. | 0,4026949 | 0,00278606 | 241,0955 | 598,7053 | 0 | 0 |
| 11799 | Birc5 | Mus musculus baculoviral IAP repeat-containing 5, mRNA | 0,4039335 | 0,02441479 | 298,851 | 739,8519 | 0 | 0 |
| 13849 | Ephx1 | Mus musculus epoxide hydrolase 1, microsomal (Ephx1), mRNA. | 0,4056903 | 0,00040758 | 537,3201 | 1324,459 | 0 | 0 |
| 19366 | Rad54l | Mus musculus RAD54 like (S. cerevisiae) (Rad54l), mRNA. | 0,408526 | 0,00215392 | 154,6182 | 378,4782 | 0 | 0 |
| 623474 | E130016E03Rik | Mus musculus RIKEN cDNA E130016E03 gene (E130016E03Rik), mRNA. | 0,4122388 | 0,00012304 | 226,6472 | 549,7959 | 0 | 0 |
| 319170 | Hist1h2an | Mus musculus histone cluster 1, H2an (Hist1h2an), mRNA. | 0,4216323 | 0,00745368 | 1438,155 | 3410,922 | 0 | 0 |
| 12236 | Bub1b | Mus musculus budding uninhibited by benzimidazoles 1 homolog, beta (S. cerevisiae) (Bub1b), mRNA. | 0,4261461 | 0,02058327 | 56,89853 | 133,5188 | 0 | 0 |
| 29870 | Gtse1 | Mus musculus G two S phase expressed protein 1 (Gtse1), mRNA. | 0,4277738 | 0,02097678 | 110,7062 | 258,796 | 0,03109815 | 0 |
| 71924 | Tube1 | Mus musculus epsilon-tubulin 1 (Tube1), mRNA. | 0,43468 | 0,0367394 | 54,11363 | 124,4907 | 0 | 0 |
| 233406 | Prc1 | Mus musculus protein regulator of cytokinesis 1 (Prc1), mRNA. | 0,4382378 | 9,732E-05 | 1676,238 | 3824,95 | 0 | 0 |
| 17218 | Mcm5 | Mus musculus minichromosome maintenance deficient 5, cell division cycle 46 (S. cerevisiae) (Mcm5), mRNA. | 0,4425378 | 0,00017888 | 701,3351 | 1584,803 | 0 | 0 |
| 14156 | Fen1 | Mus musculus flap structure specific endonuclease 1 (Fen1), mRNA. | 0,4502069 | 0,00027064 | 600,7226 | 1334,326 | 0 | 0 |
| 18817 | Plk1 | Mus musculus polo-like kinase 1 (Drosophila) (Plk1), mRNA. | 0,451235 | 0,02118297 | 328,1352 | 727,1936 | 0 | 0 |
| 66570 | Cenpm | Mus musculus centromere protein M (Cenpm), transcript variant 2, mRNA. | 0,4590164 | 0,02972697 | 98,11875 | 213,7587 | 0 | 0 |
| 107995 | Cdc20 | Mus musculus cell division cycle 20 homolog (S. cerevisiae) (Cdc20), mRNA. | 0,4606762 | 0,01475741 | 356,6465 | 774,1804 | 0,00026605 | 0 |
| 78658 | Ncapd3 | Mus musculus non-SMC condensin II complex, subunit D3 (Ncapd3), mRNA. | 0,4662049 | 0,00308963 | 148,381 | 318,2742 | 0 | 0 |
| 110033 | Kif22 | Mus musculus kinesin family member 22 (Kif22), mRNA. | 0,4670724 | 0,0157349 | 305,0575 | 653,1268 | 0 | 0 |
| 18969 | Pola2 | Mus musculus polymerase (DNA directed), alpha 2 (Pola2), mRNA. | 0,4673352 | 0,00330332 | 145,959 | 312,322 | 0 | 0 |
| 17313 | Mgp | Mus musculus matrix Gla protein (Mgp), mRNA. | 0,4686378 | 0,00055185 | 1551,55 | 3310,766 | 0 | 0 |
| 319169 | Hist1h2ak | Mus musculus histone cluster 1, H2ak (Hist1h2ak), mRNA. | 0,4743079 | 0,03465895 | 1917,268 | 4042,242 | 0 | 0 |
| 319167 | Hist1h2ag | Mus musculus histone cluster 1, H2ag (Hist1h2ag), mRNA. | 0,475072 | 0,00252976 | 196,9322 | 414,5313 | 0 | 0 |
| 18971 | Pold1 | Mus musculus polymerase (DNA directed), delta 1, catalytic subunit (Pold1), mRNA. | 0,4752148 | 0,01101476 | 203,1387 | 427,4671 | 0 | 0 |
| 20133 | Rrm1 | Mus musculus ribonucleotide reductase M1 (Rrm1), mRNA. | 0,4808031 | 0,00234098 | 241,6364 | 502,5684 | 0 | 0 |
| 51788 | H2afz | Mus musculus H2A histone family, member Z (H2afz), mRNA. | 0,483948 | 0,00108082 | 1990,058 | 4112,132 | 0 | 0 |
| 227929 | Cytip | Mus musculus cytohesin 1 interacting protein (Cytip), mRNA. | 0,4853653 | 0,0367394 | 183,7033 | 378,4846 | 0 | 0 |
| 57875 | Angptl4 | Mus musculus angiopoietin-like 4 (Angptl4), mRNA. | 0,4873511 | 0,00100786 | 1419,844 | 2913,389 | 0 | 0 |
| 20878 | Aurka | Mus musculus aurora kinase A (Aurka), mRNA. | 0,4881167 | 0,00285036 | 354,0324 | 725,3027 | 0 | 0 |
| 208628 | Kntc1 | Mus musculus kinetochore associated 1 (Kntc1), mRNA. | 0,4890712 | 0,01553217 | 226,2408 | 462,5927 | 0 | 0 |
| 106582 | Nrm | Mus musculus nurim (nuclear envelope membrane protein) (Nrm), mRNA. | 0,4895593 | 0,00186785 | 429,1425 | 876,5894 | 0 | 0 |
| 14793 | Cdca3 | Mus musculus cell division cycle associated 3 (Cdca3), mRNA. | 0,4896118 | 0,00202473 | 337,6298 | 689,5866 | 0 | 0 |
| 18813 | Pa2g4 | Mus musculus proliferation-associated 2G4 (Pa2g4), mRNA. | 0,4902437 | 0,00279207 | 277,7408 | 566,5363 | 0 | 0 |
| 12615 | Cenpa | Mus musculus centromere protein A (Cenpa), mRNA. | 0,4907956 | 0,00100786 | 3031,51 | 6176,727 | 0 | 0 |
| 71819 | Kif23 | Mus musculus kinesin family member 23 (Kif23), mRNA. | 0,4919041 | 0,00919945 | 149,3366 | 303,5888 | 0 | 0 |
| 67177 | Cdt1 | Mus musculus chromatin licensing and DNA replication factor 1 (Cdt1), mRNA. | 0,4935845 | 0,00215392 | 433,0677 | 877,3931 | 0 | 0 |
| 70454 | Cenpl | Mus musculus centromere protein L (Cenpl), mRNA. | 0,4961976 | 0,01783202 | 242,4204 | 488,5563 | 0 | 0 |
| 233876 | Hirip3 | Mus musculus HIRA interacting protein 3 (Hirip3), mRNA. | 0,4962093 | 0,0032575 | 297,5378 | 599,6215 | 0 | 0 |
| 243912 | Hspb6 | Mus musculus heat shock protein, alpha-crystallin-related, B6 (Hspb6), mRNA. | 0,4986427 | 0,01785002 | 308,921 | 619,5239 | 0 | 0 |
| 72155 | 2610510J17Rik | Mus musculus RIKEN cDNA 2610510J17 gene (2610510J17Rik), mRNA. | 0,506144 | 0,04592059 | 216,1187 | 426,9905 | 0 | 0 |
| 68067 | 3010026O09Rik | Mus musculus RIKEN cDNA 3010026O09 gene (3010026O09Rik), mRNA. | 0,5088252 | 0,03254513 | 119,3293 | 234,5192 | 0 | 0 |
| 110749 | Chaf1b | Mus musculus chromatin assembly factor 1, subunit B (p60) (Chaf1b), mRNA. | 0,5095858 | 0,00822713 | 233,4124 | 458,0435 | 0 | 0 |
| 20194 | S100a10 | Mus musculus S100 calcium binding protein A10 (calpactin) (S100a10), mRNA. | 0,5105466 | 0,00417779 | 408,3567 | 799,8422 | 0 | 0 |
| 66578 | 2610039C10Rik | Mus musculus RIKEN cDNA 2610039C10 gene (2610039C10Rik), mRNA. | 0,5123274 | 0,00441341 | 408,5093 | 797,3599 | 0 | 0 |
| 319171 | Hist1h2ao | Mus musculus histone cluster 1, H2ao (Hist1h2ao), mRNA. | 0,5182117 | 0,01103901 | 8889,065 | 17153,35 | 0 | 0 |
| 12443 | Ccnd1 | Mus musculus cyclin D1 (Ccnd1), mRNA. | 0,5437478 | 0,02335521 | 502,4753 | 924,0963 | 0 | 0 |
| 16319 | Incenp | Mus musculus inner centromere protein (Incenp), mRNA. | 0,5458675 | 0,03154558 | 255,6645 | 468,3636 | 0 | 0 |
| 319176 | Hist2h2ac | Mus musculus histone cluster 2, H2ac (Hist2h2ac), mRNA. | 0,5472458 | 0,02118297 | 7429,476 | 13576,12 | 0 | 0 |
| 24059 | Slco2a1 | Mus musculus solute carrier organic anion transporter family, member 2a1 (Slco2a1), mRNA. | 0,5490026 | 0,04423893 | 204,7135 | 372,8825 | 0 | 0 |
| 227613 | Tubb2c | Mus musculus tubulin, beta 2c (Tubb2c), mRNA. | 0,5553107 | 0,01659274 | 1168,16 | 2103,615 | 0 | 0 |
