# Supplementary material for: Growth Factor PDGF-BB Stimulates Cultured Cardiomyocytes to Synthesize the Extracellular Matrix Component Hyaluronan
Source: PLoS One. 2010 Dec 21;5(12):e14393. doi: 10.1371/journal.pone.0014393 (PMC3006157; doi:10.1371/journal.pone.0014393)
Supplement: Table S3 — Differentially expressed genes in cardiomyocytes cultured in medium with added native-HA. (0.07 MB DOC) [file pone.0014393.s003.doc]

| Table S3. Differentially expressed genes in cardiomyocytes cultured in medium with added native-HA. | | | | | | | | |
| --- | --- | --- | --- | --- | --- | --- | --- | --- |
| ENTREZ GENE ID | SYMBOL | DEFINITION | Foldchange | Diff *P*-value | Treated cells average signal | Control average signal | Treated cells detection *P*-value | Control detection *P*-value |
| 56462 | Mtch1 | Mus musculus mitochondrial carrier homolog 1 (C. elegans) (Mtch1), nuclear gene encoding  mitochondrial protein, mRNA. | 2,662898 | 0,04111822 | 692,5428 | 260,0711 | 1,96293E-05 | 0 |
| 19043 | Ppm1b | Mus musculus protein phosphatase 1B, magnesium dependent, beta isoform (Ppm1b), mRNA.  XM_925494 XM_925495 XM_925496 | 1,642929 | 0,00197373 | 445,8544 | 271,3777 | 0,0027033 | 0 |
| 26378 | Decr2 | Mus musculus 2-4-dienoyl-Coenzyme A reductase 2, peroxisomal, mRNA | 0,1748769 | 0,01699051 | 28,06553 | 160,4873 | 6,35989E-05 | 0 |
| 109900 | Asl | Mus musculus argininosuccinate lyase, mRNA | 0,1872649 | 0,00036149 | 15,12609 | 80,77374 | 0,001520091 | 0 |
| 233905 | Zfp646 | Mus musculus zinc finger protein 646 (Zfp646), mRNA. | 0,1898022 | 3,1858E-05 | 14,46821 | 76,22781 | 0,03132832 | 0 |
| 76740 | Efr3a | Mus musculus EFR3 homolog A (S. cerevisiae) (Efr3a), mRNA. | 0,2057509 | 0,00371429 | 40,19604 | 195,3627 | 0,002506266 | 0 |
| 24067 | Srp54 | Mus musculus signal recognition particle 54 (Srp54), mRNA. | 0,210789 | 0,01432766 | 44,78708 | 212,4735 | 0,002506266 | 0 |
| 105663 | Thtpa | Mus musculus thiamine triphosphatase (Thtpa), mRNA. | 0,2168116 | 0,00028741 | 11,72906 | 54,09797 | 0,04636591 | 0 |
| 50496 | E2f6 | Mus musculus E2F transcription factor 6 (E2f6), mRNA. | 0,2202784 | 0,04916262 | 29,36172 | 133,2937 | 0,003169413 | 0,000656072 |
| 84585 | Rnf123 | Mus musculus ring finger protein 123 (Rnf123), mRNA. | 0,2214333 | 0,00617959 | 18,20774 | 82,22677 | 0,01879699 | 0 |
| 19047 | Ppp1cc | Mus musculus protein phosphatase 1, catalytic subunit, gamma isoform (Ppp1cc), mRNA. | 0,2248195 | 0,01328666 | 20,64627 | 91,83484 | 0,01378446 | 0 |
| 18604 | Pdk2 | Mus musculus pyruvate dehydrogenase kinase, isoenzyme 2 (Pdk2), mRNA. | 0,2322057 | 0,00534118 | 11,94879 | 51,45778 | 0,03884712 | 0 |
| 56030 | Tmem131 | Mus musculus transmembrane protein 131 (Tmem131), mRNA. | 0,2374631 | 0,02120049 | 61,2659 | 258,0018 | 0 | 0 |
| 59035 | Carm1 | Mus musculus coactivator-associated arginine methyltransferase 1 (Carm1), mRNA. | 0,2409295 | 0,00033249 | 23,48982 | 97,49665 | 0,006265664 | 0 |
| 66251 | Arfgap3 | Mus musculus ADP-ribosylation factor GTPase activating protein 3 (Arfgap3), mRNA. | 0,2436517 | 0,00227486 | 13,11465 | 53,82538 | 0,03508772 | 0 |
| 12531 | Cdc25b | Mus musculus cell division cycle 25 homolog B (S. pombe) (Cdc25b), mRNA. | 0,2463148 | 0,0030249 | 19,19408 | 77,92498 | 0,01378446 | 0 |
| 19301 | Pxmp2 | Mus musculus peroxisomal membrane protein 2 (Pxmp2), mRNA. | 0,2521211 | 0,00255713 | 37,99694 | 150,7091 | 0,002506266 | 0 |
| 227960 | Gca | Mus musculus grancalcin (Gca), mRNA. | 0,2597008 | 0,02183136 | 16,44883 | 63,3376 | 0,00865651 | 0,000176664 |
| 17691 | Snf1lk | Mus musculus SNF1-like kinase (Snf1lk), mRNA. | 0,276773 | 0,0266811 | 85,89746 | 310,3535 | 0 | 0 |
| 20227 | Sart1 | Mus musculus squamous cell carcinoma antigen recognized by T-cells 1 (Sart1), mRNA. | 0,2809173 | 0,0006633 | 102,9318 | 366,4131 | 0 | 0 |
| 114774 | Pawr | Mus musculus PRKC, apoptosis, WT1, regulator (Pawr), mRNA. | 0,2888234 | 0,00487112 | 33,84114 | 117,169 | 0,003759399 | 0 |
| 70012 | Ccdc21 | Mus musculus coiled-coil domain containing 21 (Ccdc21), mRNA. | 0,3016791 | 0,00801101 | 23,34778 | 77,39277 | 0,006265664 | 0 |
| 72454 | Ccdc71 | Mus musculus coiled-coil domain containing 71 (Ccdc71), mRNA. | 0,302595 | 0,02545708 | 17,02743 | 56,27137 | 0,02005013 | 0 |
| 403187 | D630048P19Rik | Mus musculus RIKEN cDNA D630048P19Rik gene (D630048P19Rik), mRNA | 0,3303602 | 0,03929179 | 53,64832 | 162,3934 | 0 | 0 |
| 214469 | Fam168b | Mus musculus family with sequence similarity 168, member B (Fam168b), mRNA. | 0,3460164 | 0,01170796 | 77,5709 | 224,1827 | 0 | 0 |
| 72549 | Reep4 | Mus musculus receptor accessory protein 4 (Reep4), mRNA. | 0,3514102 | 0,0214466 | 144,8313 | 412,1432 | 0 | 0 |
| 67087 | Ctnnbip1 | Mus musculus catenin beta interacting protein 1 (Ctnnbip1), mRNA. | 0,3597143 | 0,0252362 | 25,78571 | 71,68386 | 0,005012531 | 0 |
| 58523 | Statip1 | Mus musculus signal transducer and activator of transcription interacting protein 1 (Statip1), mRNA. | 0,3605922 | 0,00175125 | 60,8965 | 168,8791 | 0 | 0 |
| 24100 | Tpra40 | Mus musculus transmembrane protein, adipocyte asscociated 1, mRNA | 0,3662225 | 0,00175125 | 33,38589 | 91,16287 | 0,003759399 | 0 |
| 15258 | Hipk2 | Mus musculus homeodomain interacting protein kinase 2 (Hipk2), mRNA. | 0,4035766 | 0,00855682 | 35,67634 | 88,40042 | 0,002506266 | 0 |
| 50798 | Gne | Mus musculus glucosamine (Gne), mRNA. | 0,4216904 | 0,00829542 | 44,45829 | 105,4287 | 0,001253133 | 0 |
| 17868 | Mybpc3 | Mus musculus myosin binding protein C, cardiac (Mybpc3), mRNA. | 0,4249018 | 0,02573116 | 787,1401 | 1852,523 | 0 | 0 |
| 13498 | Atn1 | Mus musculus atrophin 1 (Atn1), mRNA. | 0,4590715 | 0,04234785 | 46,34561 | 100,9551 | 0,001253133 | 0 |
| 13808 | Eno3 | Mus musculus enolase 3, beta muscle (Eno3), mRNA. | 0,4867543 | 0,00881986 | 367,8353 | 755,6898 | 0 | 0 |
| 100044324 | LOC100044324 | PREDICTED: Mus musculus similar to euchromatic histone methyltransferase 1 (LOC100044324), mRNA. | 0,604021 | 1,78E-09 | 56,57972 | 93,67177 | 0,01838007 | 0 |
