# Supplementary material for: Growth Factor PDGF-BB Stimulates Cultured Cardiomyocytes to Synthesize the Extracellular Matrix Component Hyaluronan
Source: PLoS One. 2010 Dec 21;5(12):e14393. doi: 10.1371/journal.pone.0014393 (PMC3006157; doi:10.1371/journal.pone.0014393)
Supplement: Table S4 — Differentially expressed genes in cardiomyocytes cultured in medium with added oligo-HA. (0.11 MB DOC) [file pone.0014393.s004.doc]

| Table S4. Differentially expressed genes in cardiomyocytes cultured in medium with added oligo-HA. | | | | | | | | |
| --- | --- | --- | --- | --- | --- | --- | --- | --- |
| ENTREZ GENE ID | SYMBOL | DEFINITION | Foldchange | Diff *P*-value | Treated cells average signal | Control average signal | Treated cells detection *P*-value | Control detection *P*-value |
| 66168 | Grina | glutamate receptor, ionotropic, N-methyl D-aspartate-associated protein 1  (glutamate binding) (Grina), mRNA. | 2,243258 | 1,508E-10 | 220,2892 | 98,2006 | 0 | 0 |
| 12028 | Bax | Bcl2-associated X protein (Bax), mRNA. | 2,231754 | 1,9121E-06 | 180,5016 | 80,87881 | 0 | 0 |
| 19703 | Renbp | renin binding protein (Renbp), mRNA. | 2,22272 | 0,00044281 | 174,8653 | 78,67175 | 0 | 0 |
| 18158 | Nppb | natriuretic peptide precursor type B (Nppb), mRNA. | 2,076041 | 0,00826121 | 156,8161 | 75,53615 | 0 | 0 |
| 235043 | Tmem205 | transmembrane protein 205 (Tmem205), mRNA. | 1,975246 | 0,00339854 | 71,44855 | 36,17199 | 0 | 0 |
| 56700 | 0610031J06Rik | RIKEN cDNA 0610031J06 gene (0610031J06Rik), mRNA. | 1,97272 | 0,00290458 | 87,62656 | 44,41917 | 0,001063225 | 0,008855311 |
| 60406 | Sap30 | sin3 associated polypeptide (Sap30), mRNA. | 1,930471 | 3,1063E-06 | 150,9759 | 78,20678 | 0 | 0 |
| 66085 | Eif3f | eukaryotic translation initiation factor 3, subunit F (Eif3f), mRNA. | 1,926809 | 4,2437E-10 | 388,2432 | 201,4954 | 0 | 0 |
| 11898 | Ass1 | argininosuccinate synthetase 1 (Ass1), mRNA. | 1,916057 | 1,7761E-08 | 245,2007 | 127,9715 | 0 | 0 |
| 12450 | Ccng1 | cyclin G1 (Ccng1), mRNA. | 1,890647 | 0,00202976 | 469,3126 | 248,2286 | 0,002584636 | 0,01074707 |
| 20115 | Rps7 | ribosomal protein S7 (Rps7), mRNA. | 1,802353 | 9,4945E-05 | 4176,617 | 2317,313 | 0 | 0 |
| 20054 | Rps15 | ribosomal protein S15 (Rps15), mRNA. | 1,789768 | 0,00020625 | 119,8732 | 66,97694 | 0 | 0 |
| 212647 | Aldh4a1 | aldehyde dehydrogenase 4 family, member A1 (Aldh4a1), nuclear gene encoding  mitochondrial protein, mRNA. | 1,771253 | 4,7656E-06 | 411,5978 | 232,3766 | 0 | 0 |
| 12848 | Cops2 | COP9 (constitutive photomorphogenic) homolog, subunit 2 (Arabidopsis thaliana) (Cops2), mRNA. | 1,770503 | 0,04214247 | 105,193 | 59,41419 | 0 | 0 |
| 77836 | Mlana | melan-A, mRNA | 1,724729 | 5,2256E-09 | 2157,421 | 1250,875 | 0 | 0 |
| 216190 | Appl2 | adaptor protein, phosphotyrosine interaction, PH domain and leucine zipper containing 2 (Appl2), mRNA. | 1,694809 | 0,00150239 | 292,8642 | 172,8007 | 0 | 0 |
| 69554 | Klhdc2 | kelch domain containing 2 (Klhdc2), mRNA. | 1,677104 | 2,0127E-06 | 330,4191 | 197,0177 | 0 | 0 |
| 216395 | Tmem5 | transmembrane protein 5 (Tmem5), mRNA. | 1,669156 | 0,00779426 | 100,921 | 60,46226 | 0 | 0 |
| 64660 | Mrps24 | mitochondrial ribosomal protein S24 (Mrps24), nuclear gene encoding mitochondrial protein, mRNA. | 1,655201 | 0,0022656 | 118,5729 | 71,63655 | 0 | 0 |
| 102032 | AI316807 | expressed sequence AI316807 (AI316807), mRNA. | 1,635679 | 0,0048832 | 153,0273 | 93,55582 | 0 | 0 |
| 100340 | Smpdl3b | sphingomyelin phosphodiesterase, acid-like 3B (Smpdl3b), mRNA. | 1,635221 | 0,00518964 | 262,6915 | 160,6459 | 0 | 0 |
| 13014 | Cstb | cystatin B (Cstb), mRNA. | 1,634513 | 0,00024625 | 2485,505 | 1520,64 | 0 | 0 |
| 11972 | Atp6v0d1 | ATPase, H+ transporting, lysosomal V0 subunit D1 (Atp6v0d1), mRNA. | 1,626006 | 0,01820409 | 219,6273 | 135,0716 | 0,000462938 | 0,001188133 |
| 67097 | Rps10 | ribosomal protein S10 (Rps10), mRNA. | 1,62301 | 0,00023934 | 178,3634 | 109,8967 | 0 | 0 |
| 67264 | Ndufb8 | NADH dehydrogenase (ubiquinone) 1 beta subcomplex 8 (Ndufb8), mRNA. | 1,618068 | 0,00021276 | 318,8579 | 197,0609 | 0 | 0 |
| 214505 | Gnptg | N-acetylglucosamine-1-phosphotransferase, gamma subunit (Gnptg), mRNA. | 1,606955 | 0,02171613 | 162,2872 | 100,9905 | 0 | 0 |
| 72657 | 2700094K13Rik | RIKEN cDNA 2700094K13 gene (2700094K13Rik), transcript variant 2, mRNA. | 1,604514 | 0,00021413 | 410,5582 | 255,877 | 0 | 0 |
| 231946 | D330028D13Rik | RIKEN cDNA D330028D13 gene (D330028D13Rik), mRNA. | 1,597687 | 0,0022656 | 155,7366 | 97,47634 | 0 | 0 |
| 214917 | Fam173a | family with sequence similarity 173, member A (Fam173a), mRNA. | 1,597593 | 0,04025927 | 85,63089 | 53,59995 | 0 | 0 |
| 26360 | Angptl2 | angiopoietin-like 2 (Angptl2), mRNA. | 1,576551 | 0,03344444 | 105,8262 | 67,12514 | 0 | 0 |
| 26362 | Axl | AXL receptor tyrosine kinase (Axl), mRNA. | 1,567711 | 0,03998666 | 201,6957 | 128,6562 | 0 | 0 |
| 78100 | 8430410K20Rik | RIKEN cDNA 8430410K20 gene (8430410K20Rik), mRNA. | 1,567086 | 6,8335E-05 | 309,2428 | 197,3362 | 0 | 0 |
| 73010 | Gpr22 | G protein-coupled receptor 22 (Gpr22), mRNA. | 1,563349 | 0,04812873 | 107,1716 | 68,55254 | 0 | 0 |
| 675813 | LOC675813 | PREDICTED: similar to ribosomal protein S20 (LOC675813), misc RNA. | 1,560068 | 0,00109343 | 183,711 | 117,7584 | 0 | 0 |
| 30057 | Timm8b | translocase of inner mitochondrial membrane 8 homolog b (yeast) (Timm8b), mRNA. | 1,547022 | 0,03757548 | 116,1262 | 75,06438 | 0 | 3,84734E-05 |
| 15312 | Hmgn1 | high mobility group nucleosomal binding domain 1 (Hmgn1), mRNA. | 1,534024 | 0,01992167 | 247,8082 | 161,5413 | 0 | 0 |
| 56448 | Cyp2d22 | cytochrome P450, family 2, subfamily d, polypeptide 22 (Cyp2d22), mRNA. | 1,511627 | 0,01361952 | 132,4227 | 87,60275 | 0 | 0 |
| 17691 | Snf1lk | SNF1-like kinase (Snf1lk), mRNA. | 0,419512 | 2,193E-27 | 130,1968 | 310,3535 | 0 | 0 |
| 76130 | Las1l | LAS1-like (S. cerevisiae) (Las1l), mRNA. | 0,434162 | 0,0002786 | 30,95399 | 71,29596 | 0 | 0 |
| 21924 | Tnnc1 | troponin C, cardiac/slow skeletal (Tnnc1), mRNA. | 0,469362 | 1,8403E-16 | 1083,17 | 2307,748 | 0 | 0 |
| 17897 | Myl3 | myosin, light polypeptide 3 (Myl3), mRNA. | 0,496082 | 0,0092326 | 31,23584 | 62,9651 | 0 | 0 |
| 16322 | Inha | inhibin alpha (Inha), mRNA. | 0,498399 | 0,03955415 | 32,84009 | 65,89118 | 0 | 0 |
| 19417 | Rasgrf1 | RAS protein-specific guanine nucleotide-releasing factor 1 (Rasgrf1), transcript variant 1, mRNA. | 0,541531 | 4,0417E-05 | 96,18937 | 177,625 | 0,000990963 | 0,004484062 |
| 56437 | Rrad | Ras-related associated with diabetes, mRNA | 0,542855 | 8,837E-17 | 300,7486 | 554,0132 | 0 | 0 |
| 12531 | Cdc25b | cell division cycle 25 homolog B (S. pombe) (Cdc25b), mRNA. | 0,57858 | 0,00541943 | 45,08586 | 77,92498 | 0 | 0 |
| 66402 | Sln | sarcolipin (Sln), mRNA. | 0,588039 | 2,8325E-05 | 75,34833 | 128,1348 | 0 | 0 |
| 22668 | Sf1 | splicing factor 1 (Sf1), mRNA. XM_979946 XM_979979 | 0,591686 | 0,04876193 | 44,47563 | 75,16756 | 0,001885195 | 0,0122683 |
| 100048083 | LOC100048083 | PREDICTED: similar to Tect2 (LOC100048083), misc RNA. | 0,595856 | 0,00040607 | 73,45003 | 123,268 | 0 | 0 |
| 18611 | Pea15 | phosphoprotein enriched in astrocytes, mRNA | 0,606383 | 1,1327E-09 | 223,3682 | 368,3613 | 0 | 0 |
| 26378 | Decr2 | 2-4-dienoyl-Coenzyme A reductase 2, peroxisomal, mRNA | 0,607146 | 0,00022363 | 97,43923 | 160,4873 | 0 | 0 |
| 50496 | E2f6 | E2F transcription factor 6 (E2f6), mRNA. | 0,608898 | 0,01682822 | 81,16222 | 133,2937 | 0,001849695 | 0,000656072 |
| 70399 | 2310058J06Rik | RIKEN cDNA 2310058J06Rik gene (2310058J06Rik), mRNA | 0,610741 | 0,0092326 | 118,543 | 194,0971 | 0 | 0 |
| 114774 | Pawr | PRKC, apoptosis, WT1, regulator (Pawr), mRNA. | 0,611995 | 0,00040607 | 71,70677 | 117,169 | 0 | 0 |
| 15258 | Hipk2 | homeodomain interacting protein kinase 2 (Hipk2), mRNA. | 0,614346 | 0,00763148 | 54,30846 | 88,40042 | 0 | 0 |
| 19301 | Pxmp2 | peroxisomal membrane protein 2 (Pxmp2), mRNA. | 0,615928 | 0,00268515 | 92,82585 | 150,7091 | 0 | 0 |
| 19349 | Rab7 | RAB7, member RAS oncogene family (Rab7), mRNA. | 0,626922 | 0,00080765 | 165,736 | 264,3645 | 0,003305227 | 0,001084923 |
| 21345 | Tagln | transgelin (Tagln), mRNA. | 0,627109 | 0,03282453 | 49,15868 | 78,38937 | 0 | 0 |
| 13992 | Khdrbs3 | KH domain containing, RNA binding, signal transduction associated 3 (Khdrbs3), mRNA. | 0,630519 | 6,0785E-08 | 229,2347 | 363,5653 | 0 | 0 |
| 18824 | Plp2 | proteolipid protein 2 (Plp2), mRNA. | 0,644358 | 6,605E-06 | 1087,949 | 1688,423 | 0 | 0 |
| 19047 | Ppp1cc | protein phosphatase 1, catalytic subunit, gamma isoform (Ppp1cc), mRNA. | 0,656118 | 0,03417668 | 60,25452 | 91,83484 | 0 | 0 |
| 72113 | Adck1 | aarF domain containing kinase 1 (Adck1), mRNA. | 0,656205 | 0,0009668 | 203,3128 | 309,8311 | 0 | 0 |
| 381101 | BC048355 | cDNA sequence BC048355, mRNA | 0,658684 | 0,0030498 | 180,9628 | 274,7339 | 0 | 0 |
| 106628 | Trip10 | thyroid hormone receptor interactor 10 (Trip10), mRNA. | 0,667933 | 0,03814778 | 63,02441 | 94,35738 | 0 | 0 |
